# Glutamatergic and GABAergic Receptor Modulation Present Unique Electrophysiological Fingerprints in a Concentration-Dependent and Region-Specific Manner

**DOI:** 10.1523/ENEURO.0406-22.2023

**Published:** 2023-04-13

**Authors:** Irene Gonzalez-Burgos, Marie Bainier, Simon Gross, Philipp Schoenenberger, José A. Ochoa, Miguel Valencia, Roger L. Redondo

**Affiliations:** 1 Roche Pharma Research and Early Development, Neuroscience and Rare Diseases, Roche Innovation Center Basel, F. Hoffmann-La Roche Ltd, Grenzacherstrasse 124, Basel 4070, Switzerland; 2 Program of Neuroscience, Centro de Investigación Médica Aplicada, Universidad de Navarra, Pamplona 31080, Spain; 3 Instituto de Investigación Sanitaria de Navarra (Navarra Institute for Health Research), Pamplona 31080, Spain; 4 Institute of Data Science and Artificial Intelligence, Universidad de Navarra, Pamplona, Spain

**Keywords:** EEG, parametrization, MK-801, diazepam, GABA, NMDA

## Abstract

Brain function depends on complex circuit interactions between excitatory and inhibitory neurons embedded in local and long-range networks. Systemic GABAA-receptor (GABAAR) or NMDA-receptor (NMDAR) modulation alters the excitatory-inhibitory balance (EIB), measurable with electroencephalography (EEG). However, EEG signatures are complex in localization and spectral composition. We developed and applied analytical tools to investigate the effects of two EIB modulators, MK801 (NMDAR antagonist) and diazepam (GABAAR modulator), on periodic and aperiodic EEG features in freely-moving male Sprague Dawley rats. We investigated how, across three brain regions, EEG features are correlated with EIB modulation. We found that the periodic component was composed of seven frequency bands that presented region-dependent and compound-dependent changes. The aperiodic component was also different between compounds and brain regions. Importantly, the parametrization into periodic and aperiodic components unveiled correlations between quantitative EEG and plasma concentrations of pharmacological compounds. MK-801 exposures were positively correlated with the slope of the aperiodic component. Concerning the periodic component, MK-801 exposures correlated negatively with the peak frequency of low-γ oscillations but positively with those of high-γ and high-frequency oscillations (HFOs). As for the power, θ and low-γ oscillations correlated negatively with MK-801, whereas mid-γ correlated positively. Diazepam correlated negatively with the knee of the aperiodic component, positively to β and negatively to low-γ oscillatory power, and positively to the modal frequency of θ, low-γ, mid-γ, and high-γ. In conclusion, correlations between exposures and pharmacodynamic effects can be better-understood thanks to the parametrization of EEG into periodic and aperiodic components. Such parametrization could be key in functional biomarker discovery.

## Visual Abstract

**Figure:**
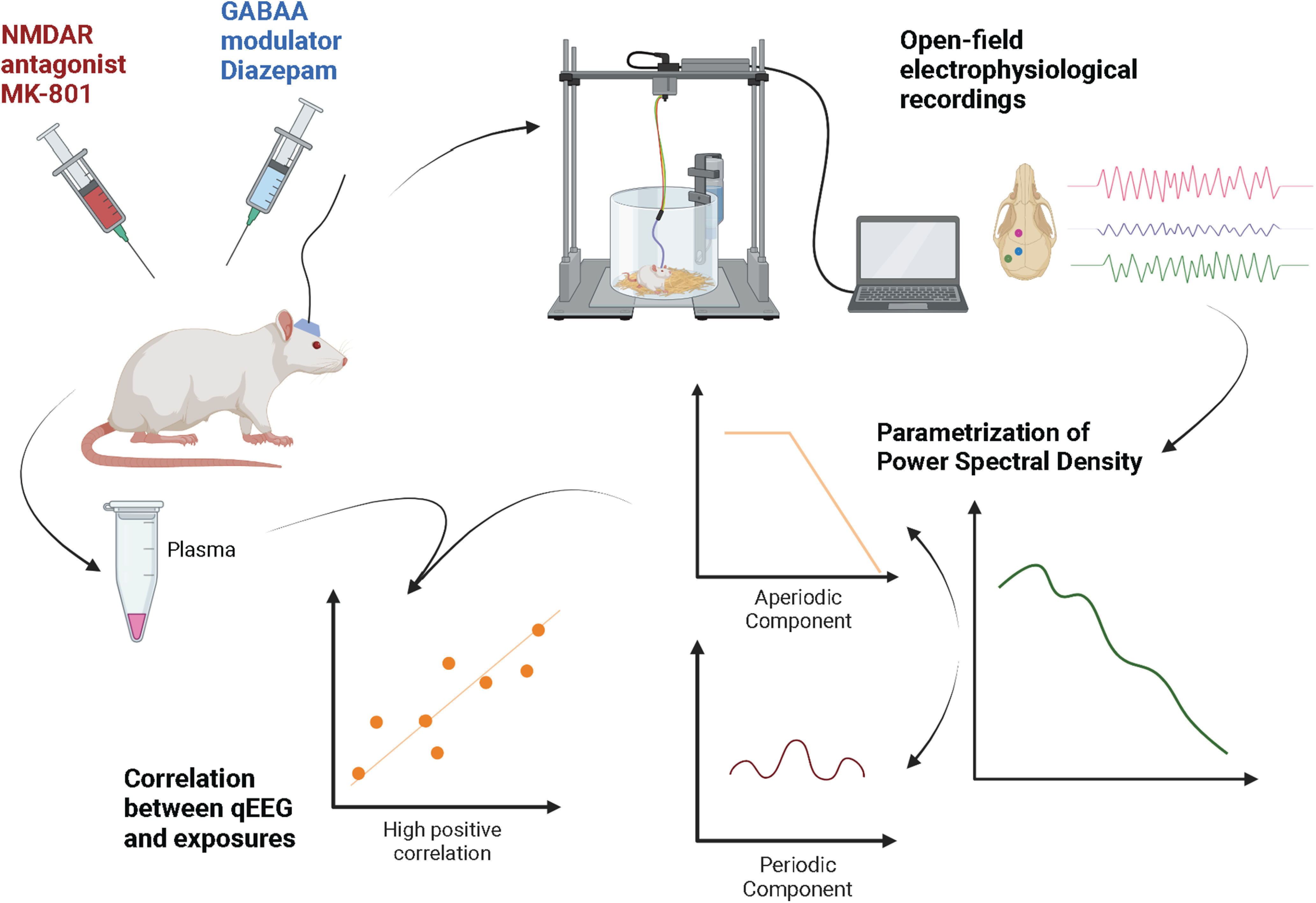

## Significance Statement

Excitatory-inhibitory balance (EIB) is compromised in neurologic disorders. Our study demonstrates that pharmacologically-induced effects on EIB can be quantified by decomposing the quantitative electroencephalography (qEEG) power spectrum signal into the oscillatory periodic and the 1/f aperiodic components. MK-801 and diazepam showed distinct signatures across brain regions and EEG components. Specific features of these components are sensitive to relatively small changes in measured exposure. This methodological approach and the features identified as sensitive to EIB modulation could be key for the development of new therapies and functional biomarkers in disorders with excitatory-inhibitory imbalance.

## Introduction

Excitatory-inhibitory balance (EIB) is a neuronal state in which excitatory and inhibitory circuits contribute to the correct functioning of neuronal networks. EIB is crucial for information processing in local microcircuits and long-range neuronal networks (Rubenstein and Merzenich, 2003). EIB can be compromised by numerous structural and functional defects of varied etiologies (Rubenstein and Merzenich, 2003). Suboptimal EIB is described in neurodevelopmental and neuropsychiatric disorders (Sohal and Rubenstein, 2019; Liu et al., 2021) and aging (Ghosh et al., 2021). The roles of Glutamatergic and GABAergic systems in EIB are evidenced through pharmacological neurotransmitter-specific modulators that induce physiological (Ebe et al., 1969; Greenblatt et al., 1989; Adler and Gattaz, 1993; Holcomb et al., 2005; Muñoz-Torres et al., 2011; De Simoni et al., 2013; Jobert and Wilson, 2015; Pflanz et al., 2015; McNally and McCarley, 2016; Walter et al., 2016; Premoli et al., 2017; Grent-’t-Jong et al., 2018; Inui et al., 2018; Uno and Coyle, 2019) and behavioral changes (Javitt and Zukin, 1991; Krystal et al., 1994; Cerne et al., 2021).

EIB is successfully monitored through electrophysiology (Liu et al., 2021). While quantitative electroencephalography (qEEG) is an invaluable tool in assessing EIB, it generates complex readouts and the interpretation is challenging. Apparent changes in power in predefined narrow frequency bands may reflect shifts in the peak frequency of the oscillation, changes in broad-band power, or changes in the aperiodic component of the spectrum (Donoghue et al., 2020). The aperiodic component of the spectrum refers to the quasi-linearly decreasing tendency of the power of the frequency spectra of qEEG signals (in log/log space; Bak, 1996; Buzsáki and Draguhn, 2004; Miller et al., 2009; Voytek et al., 2015). Canonically, descriptions of pharmacological effects have focused on the oscillatory peaks that appear over the aperiodic component: the oscillatory components of the signal. As such, GABAAR modulation through the administration of diazepam has been described to produce increases in β band oscillations (Jobert and Wilson, 2015; Premoli et al., 2017). On the other hand, NMDAR antagonism has been implicated in the appearance of γ oscillatory activity and high-frequency oscillations (HFOs; Lazarewicz et al., 2010; Frohlich and Van Horn, 2014; de la Salle et al., 2016).

Nevertheless, the aperiodic 1/f activity should not be ignored as it captures important information beyond background noise. The EEG shows a power spectrum (PS) following a 1/f pattern that reveals an intrinsic feature of many complex systems in nature (Freeman et al., 2003; Miller et al., 2009; Marković and Gros, 2014). Studies have highlighted its physiological importance, relating it to chronological age, behavioral and cognitive performance, and attention (Donoghue et al., 2020; Thuwal et al., 2021; Waschke et al., 2021). Other reports correlate it to behavioral readouts in Autism Spectrum Disorder (Wilkinson and Nelson, 2021) and dopamine depletion in Parkinson’s disease (Kim et al., 2022). Moreover, this 1/f component of the signal has been stated to reflect the EIB of neural activity (Gao et al., 2017). Optogenetic work has shown that a flattening of the slope of the aperiodic component is mechanistically linked to increased EIB (Chini et al., 2022). Increased excitation through pharmacological intervention in human studies produces a flattening of the 1/f component, such as when administered the NMDAR antagonists ketamine (Waschke et al., 2021) and memantine (Molina et al., 2020). In contrast, increased inhibition leads to a steepening of the aperiodic component, such as when administering propofol (Waschke et al., 2021). Opposite effects over the dopaminergic system (agonism/antagonism) induce opposite changes in the 1/f characteristic (Valencia et al., 2012) and serotonergic modulation decreases the 1/f slope, indicating an EIB shift in favor of excitation (Zsido et al., 2022). Questions remain about the value of such spectral decomposition in neuropharmacology, especially when applied to EIB modulators.

Here, we set out to investigate the electrophysiological responses in auditory, parietal, and frontal cortices produced by MK-801 and diazepam. We hypothesized that the opposing effects of NMDAR antagonist MK-801 and the GABAAR modulator diazepam on EIB, evidenced through the decomposition of the qEEG, would be distinct and tightly dependent on the plasma exposures (i.e., the concentration of the modulator in plasma). We tested our hypotheses by recording EEG in freely-moving awake rats after single-dose administration of MK-801 or diazepam and subsequently decomposing and parametrizing the signal into the oscillatory periodic component and the 1/f aperiodic component to correlate them with blood plasma exposures. This analysis revealed significant correlations between plasma exposures and qEEG parameters. Such novel correlations support the value of these qEEG parameters as pharmacodynamic readouts.

## Materials and Methods

### Animals

Adult male Sprague Dawley rats were used for the experiments. Animal experiments were approved by the Federal Food Safety and Veterinary Office of Switzerland and conducted in strict adherence to the Swiss federal ordinance on animal protection and welfare, as well as according to the rules of the Association for Assessment and Accreditation of Laboratory Animal Care International.

Two cohorts of animals were used. At the time of recording, animals in the first cohort (experiment 1) were young adults (69 ± 5 d old, *N* = 8), and in the second cohort (experiment 2) were older adults (147 ± 30 d old, *N* = 8). Rats were kept in a 12/12 h light/dark cycle at room temperature. Food and water were provided *ad libitum*.

### Surgical implantation of electrodes

Rats were deeply anesthetized with 4% isoflurane for 5 min in an incubation chamber. Preoperative care consisted of buprenorphine (0.2 mg/kg, s.c.) for analgesia and lidocaine-bupivacaine (0.1 ml, s.c.) as a local analgesic on the incision site. Throughout the surgery, isoflurane levels were kept at 2% – 3% using an inhalation mask. Three stainless steel screw electrodes (1.2 × 3 mm, Bossard, BN 650, ref. 1421662) were stereotactically placed in the skull perpendicular to the brain surface. Target areas were left prefrontal cortex (PFC; +2.5 AP, −1.2 ML), left parietal cortex (PAR; −4.0 AP, −3.0 ML), and left primary auditory cortex (A1; −4.8 AP, −5.5 ML). In experiment 2, two out of eight of the animals had the A1 screw placed from the side (−4.8 AP, −7.4 ML). Coordinates were calculated using (Paxinos and Watson, 2006). Screws were connected to commercial electrode drives. For experiment 1, screws were connected to Omnetics 18 Position Dual Row Male Nano-Miniature (0.025”/0.64 mm) Connector (A79020-001). Six animals of experiment 2 were implanted with Innovative Neurophysiology 16-channel movable array, and two animals were implanted with Atlas Neuroengineering 14-channel array. The type of implant did not impact the quality of the EEG data used in this study. Implants were fixed to the skull using dental (Paladur, Kulzer; Refobacin, Zimmer Biomet) and bone cement (G-CEM LinkAceTM, GC Corporation). Except for the top of the socket, the implant was covered by skin. Postoperative analgesia (Metacam, 1 mg/kg, s.c.) was administered for two consecutive days after surgery to minimize postsurgical pain. Animals were housed individually postoperatively to prevent damage to the implants.

### Experimental paradigm

Animals were habituated for multiple days to the open-field environment. Experimental sessions consisted of 15 min blocks of freely-moving open-field recordings. Two recording sessions per animal were performed each day: the first session before administering the compound (predosing/baseline session) and the second after dosing the compounds (postdosing session). Animals were subcutaneously injected (1 ml/kg) with diazepam (3 mg/kg), MK-801 (0.075 mg/kg), or vehicle (0.9% saline + 0.3% Tween 80). Postdosing sessions began 15 min after injection of diazepam or vehicle, and 30 min after injection of MK-801. The doses and postdosing recording times were chosen based on previous literature (van der Staay et al., 2011; Hurtubise et al., 2017) and our own experience, to achieve exposures that maximize physiological changes with minimal behavioral confounds. The order of testing was determined according to a Latin square design. Each animal was recorded on three different days, one per compound. Hence, all compounds were tested on all animals. No blinding was performed. The duration of the washout period between dosing was of at least 48 h.

Electrophysiological data were collected with an OpenEphys system (www.open-ephys.com) using the following settings: analog low-cut = 0.1 Hz; analog high-cut = 7600 Hz; DSP cutoff = 0.15 Hz; sampling rate = 20 kS/s. Throughout all the recording sessions, animals were videotaped. Using a custom-designed workflow in the Bonsai environment (www.bonsai-rx.org), the location coordinates of the center-of-mass of the animal within the recording box were recorded and stored for offline analysis.

After every postdosing recording session, plasma samples were immediately collected from the tail vein and stored at −80°C for posterior analyses.

### Determination of plasma concentration levels of pharmacological compounds

A qualified method was used in a specialized bioanalytical laboratory. Rat blood plasma samples (50 µl) were extracted by protein precipitation. Extracts (20 µl) were injected into the analytical column (YMC Triart C18, 3 mm; 2.1´50 mm) and analyzed using liquid chromatography coupled to tandem mass spectrometry. Separation was performed using gradient elution from 30−95% mobile phase B in 2.5 min at a flow of 0.8 ml/min, where mobile phase A was 20 mm ammonium-acetate and mobile phase B was acetonitrile. Multiple reaction monitoring was conducted on a Sciex QTrap 6500+ mass spectrometer using MRM transitions.

### Signal processing

#### Artifact removal

Both experimental groups were included in the analysis. Custom-made software in the Python environment controlling Neuroexplorer 5 software (Nex Technologies) was used to preprocess the data and detect and discard artifacts. Epochs with signals exceeding a 0.6-mV threshold for longer than 1 s were considered artifacts and excluded from the analysis (padding time: 1.5 s). Additionally, recording channels with suboptimal signal quality were excluded from further analysis.

#### Behavioral segmentation

Behavioral segmentation was performed by smoothing video-tracking data (Gaussian filter; window size: 166 ms) and synchronizing it to the electrophysiological data. Two behavioral states were defined based on the speed of the animal. Segments where the center-of-mass of the animal moved at a speed superior to 1 cm/s for at least 1 s were classified as “Moving.” The complementary segments were classified as “Still.” The “Still” segment includes both quiet-wakefulness as well as sleep states.

#### Power spectral analysis

For each animal and condition, “Still” segments were selected, and power spectra (PSs) were computed using MATLAB’s *pspectrum()* function (MathWorks). Parameters were set to estimate the frequency content of the signals between 0.1 and 190 Hz, with a spectral leakage of 0.85 and a resolution of 0.5 Hz per bin. PSs were transformed into decibels by using a logarithmic transformation. Custom-made software was employed to remove 50 Hz and its harmonic peaks.

#### Parametrization of the power spectral density

**Figure 1.**
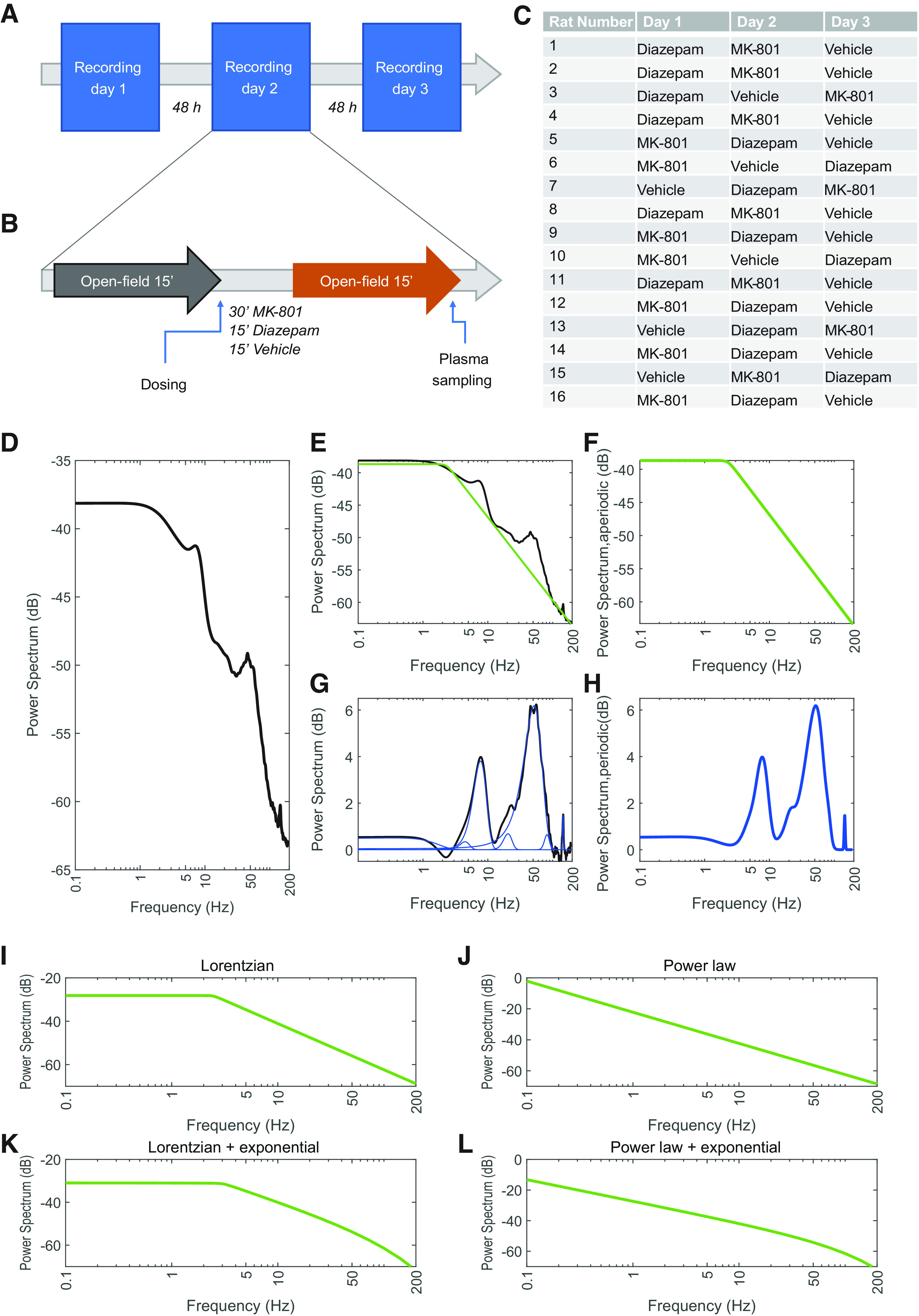
Experimental design and schematic analytic sequence. Overview of experimental timeline (***A***), experimental paradigm (***B***), and dosing scheme (***C***). Three experimental blocks with at least 48 h between each block were conducted. Each experimental block included testing of all 16 animals. Experimental blocks consisted of two 15-min sessions, one predosing, and one postdosing session. Postdosing sessions began 15 min after the administration of diazepam and vehicle, and 30 min after MK-801. Animals were injected according to the dosing scheme. Schematic decomposition of exemplary (***D***) power spectrum (PS) into its (***E***) aperiodic and (***G***) periodic components. ***E***, The PS is first fitted with an estimated aperiodic component (green). The estimated aperiodic portion of the signal (***F***) is subtracted from the raw PS, and (***E***) multi-Gaussian fitting is then performed on the residuals (blue). ***H***, The sum of the several gaussian functions constitutes the periodic component. Fitting of the PS can be performed using (***I***) Lorentzian, (***J***) power law, (***K***) Lorentzian plus exponential decay, and (***L***) power law plus exponential decay functions.

The PSs were then decomposed into their periodic and aperiodic components using custom-made software in the MATLAB environment (Fig. 1*D–H*). The PS aperiodic component was modeled using four functions: power law, Lorentzian, power-law plus exponential decay, and Lorentzian plus exponential decay (Fig. 1*I–L*). We tested goodness of fit [root-mean-square error (RMSE); Extended Data Fig. 2-1] for all of them under the different conditions and the different regions. We found that the overall best fit was provided by the Lorentzian function (*b + 1/(f^x^ + k)*), characterized by the offset (*b*), the slope (*X*), and the knee (*k*). Once the aperiodic component had been determined, the periodic component was extracted by fitting several Gaussians on the residual signal (Valencia et al., 2012; Donoghue et al., 2020).

The PS has been classically segmented into oscillatory bands because of their implication in distinct functional (e.g., γ in cognition) and physiological (e.g., α in sleep) roles. However, these bands are arbitrarily defined and no definitive consensus exists concerning their limits. To overcome this, we considered the frequency distribution of the Gaussian functions fitted to the periodic component per condition (independently of the region) and used a kernel-density estimator (KDE; width 0.05 Hz, from 0.1 to 190 Hz within 100 points) to determine the frequencies where oscillatory modes appear (Valencia et al., 2012). Frequency band limits were then defined as the inflection points in the KDE, offering a total of seven oscillatory bands: δ, θ, β, low-γ, mid-γ, high-γ, and HFO.

Within each PS, for each of the condition-specific frequency bands, the Gaussian functions encompassed within those frequency limits were reconstructed (Table 1). Out of the resulting curve, three parameters were extracted: the amplitude of the peak, the frequency at which the peak occurs (i.e., modal frequency), and the overall power [defined as the area under the curve (AUC)].

**Table 1:** Condition-specific frequency band limits as established by KDE

	Vehicle	Diazepam	MK-801
δ	<5.3	<4.6	<4.0
Θ	5.3–9.7	4.6–10.5	4.0–9.0
β	9.7–19.3	10.5–22.5	9.0–19.3
Low-γ	19.3–41.3	22.5–32.9	19.3–32.9
Mid-γ	41.3–70.5	32.9–60.5	32.9–60.5
High-γ	70.5–111.4	60.5–120.2	60.5–111.4
HFO	>111.4	>120.2	>111.4

For the three conditions studied (vehicle postdosing; diazepam postdosing; MK-801 postdosing), seven oscillations bands were identified. Shown are the frequency limits of each oscillatory band in Hz.

### Statistical analysis

**Figure 2.**
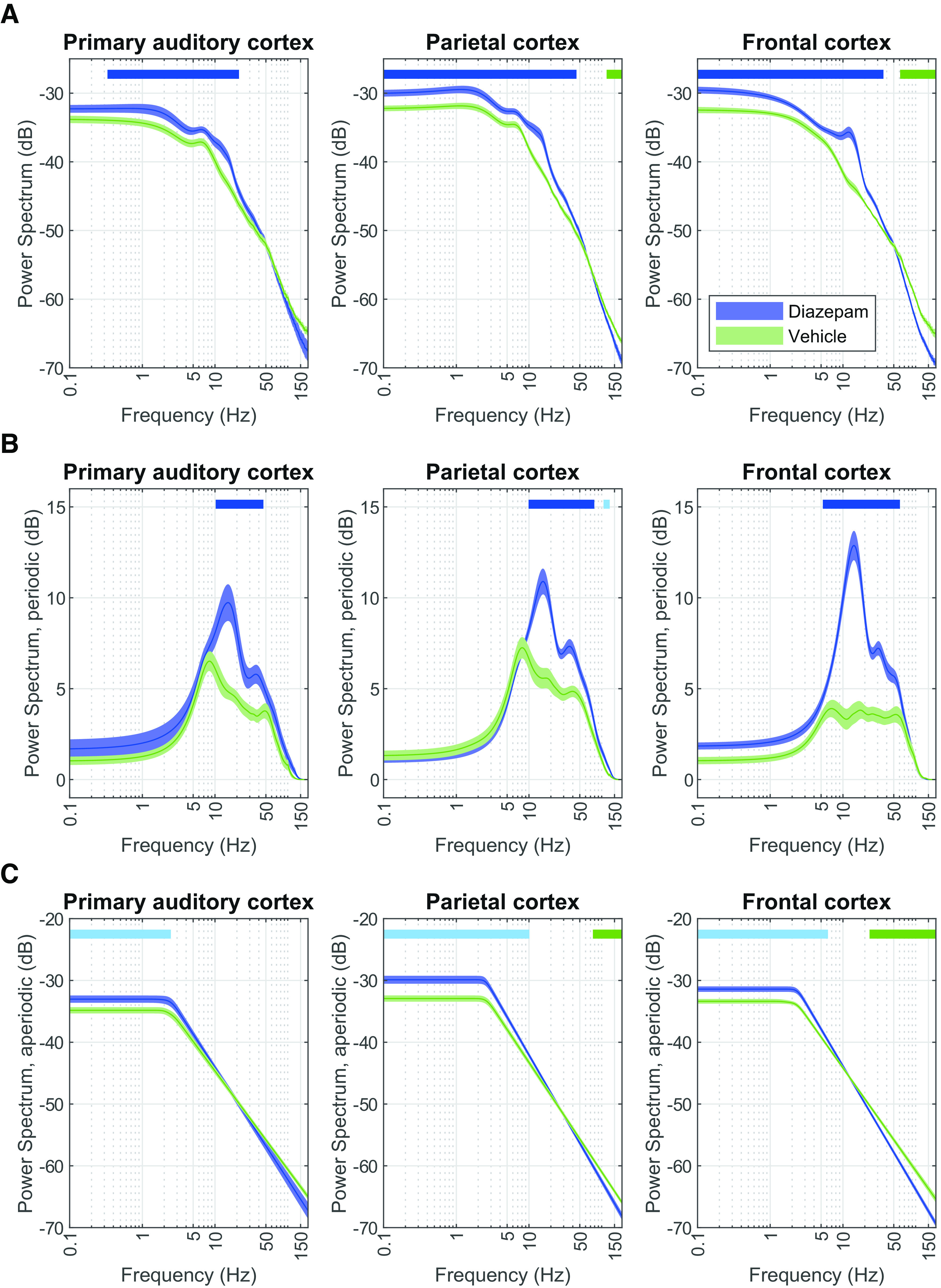
Diazepam affects the power spectrum (PS) by increasing β and γ amplitudes in the periodic component and power of low frequencies in the aperiodic. PSs (***A***), periodic component (***B***), and aperiodic component (***C***) in the primary auditory (left; *N* = 9), parietal (center, *N* = 12), and frontal cortex (right; *N* = 11) under the effects of diazepam (blue) and vehicle (green). PSD decomposition was performed by fitting a Lorentzian function, which offered the best fit for our data (Extended Data Fig. 2-1). Although there are slight effects of habituation as shown by comparing vehicle predosing and postdosing in Extended Data Figure 2-2, described effects of diazepam are similar when comparing it to the predosing session, as shown in Extended Data Figure 2-3. Solid lines indicate the average and shaded areas describe the SEM. Top horizontal bars indicate clusters identified by paired CBP analysis (lighter shade *p* < 0.01; darker shade *p* < 0.05, *N*_permutations_ = 1000).

**Figure 3.**
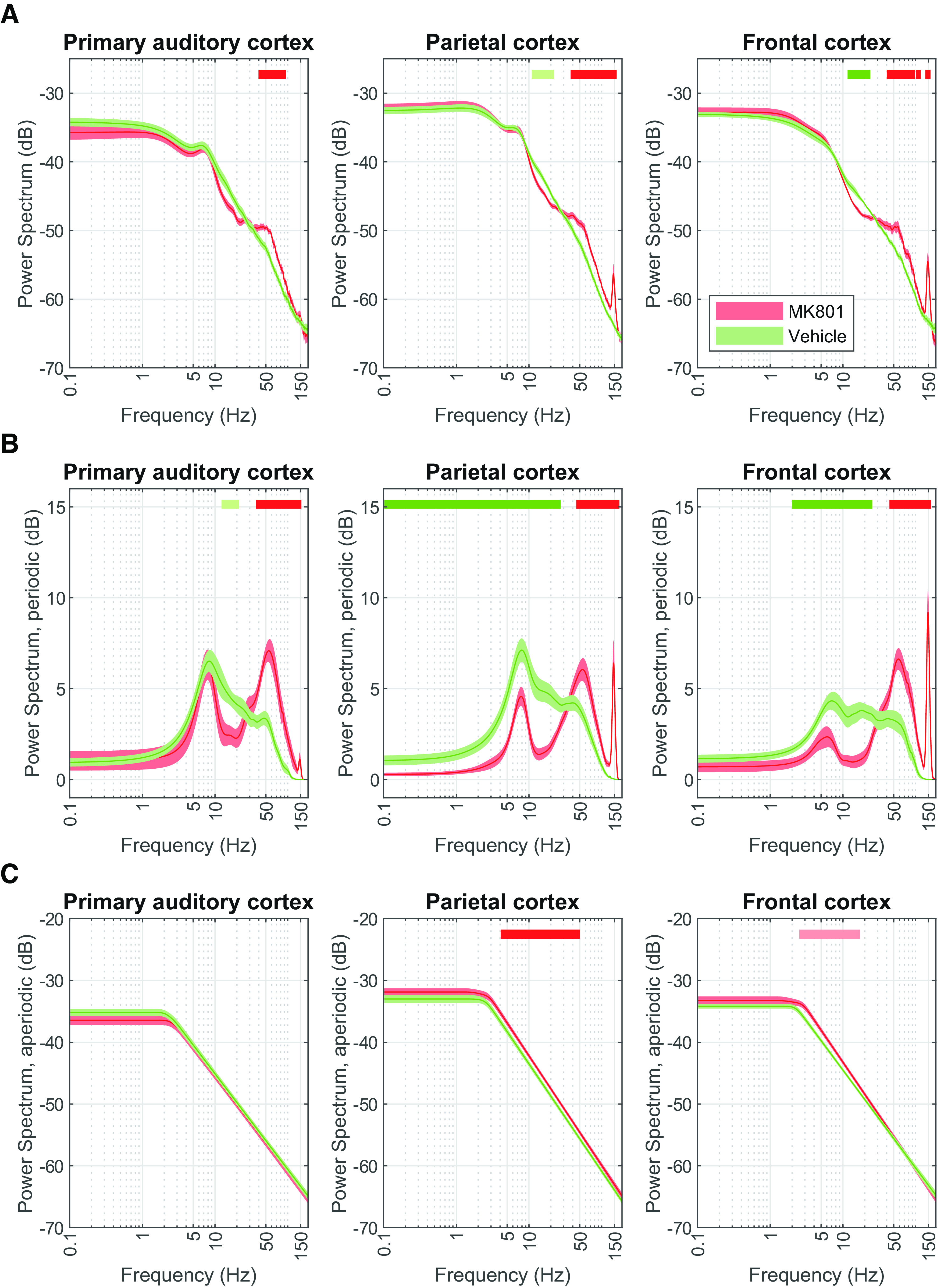
MK-801 affects the power spectrum (PS) by increasing amplitudes of higher frequencies in the periodic component and power of lower frequencies in the aperiodic. PSs (***A***), periodic component (***B***), aperiodic component (***C***) in the primary auditory (left; *N* = 7), parietal (center; *N* = 12), and frontal cortex (right; *N* = 9) under the effects of MK-801 (red) and vehicle (green). Results are replicated when comparing it to the predosing session, as shown in Extended Data Figure 3-1. Solid lines indicate the average and shaded areas describe the SEM. Top horizontal bars indicate clusters identified by paired CBP analysis (lighter shade *p* < 0.01; darker shade *p* < 0.05, *N*_permutations_ = 1000).

Statistical testing was performed using custom-made scripts in MATLAB. For PS, statistical testing was performed with a paired cluster-based permutation (CBP) test (1000 permutations, *p* < 0.05). CBP test deals with the multiple comparisons problem and allows the identification of significant clusters in continuous data, e.g., in the time or frequency domain (Nichols and Holmes, 2001; Maris and Oostenveld, 2007; Sassenhagen and Draschkow, 2019). Only animals with paired sessions were included. Qualitatively no apparent outliers were present; thus, no specific test was performed for outlier detection. Compounds were compared with both their corresponding vehicle (Figs. 2, 3) and predosing sessions (Extended Data Figs. 2-2, 2-3, 3-1).

10.1523/ENEURO.0406-22.2023.f2-1 Extended Data Figure 2-1 Root-mean-square error (RMSE) of the fit according to the fitting function used under different dosing conditions in the (***A***) primary auditory, (***B***) parietal, and (***C***) frontal cortices. Overall, the Lorentzian function offers the best goodness of fit. Download Figure 2-1, EPS file.

10.1523/ENEURO.0406-22.2023.f2-2 Extended Data Figure 2-2 Effect of dosing and habituation slightly increases γ oscillatory activity as evidenced by comparing the (***A***) power spectrum (PS), (***B***) periodic component, (***C***) aperiodic component over the primary auditory (left; *N* = 8), parietal (center; *N* = 10), and frontal cortex (right; *N* = 9) before dosing session (grey) and after dosing with vehicle session (green). Solid lines indicate the average and shaded areas describe the SEM. Top horizontal bars indicate clusters identified by paired CBP analysis (lighter shade *p* < 0.01; darker shade *p* < 0.05). Download Figure 2-2, EPS file.

10.1523/ENEURO.0406-22.2023.f2-3 Extended Data Figure 2-3 Diazepam affects the power spectrum (PS) by increasing β and γ frequencies in the periodic component and low frequencies in the aperiodic. PSs (***A***), periodic component (***B***), aperiodic component (***C***) in the primary auditory (left; *N* = 7), parietal (center; *N* = 10), and frontal cortex (right; *N* = 9) under the effects of diazepam (blue) and vehicle (green). Solid lines indicate the average and shaded areas describe the SEM. Top horizontal bars indicate clusters identified by paired CBP analysis (lighter shade *p* < 0.01; darker shade *p* < 0.05). Download Figure 2-3, EPS file.

10.1523/ENEURO.0406-22.2023.tab2-1 Extended Data Table 2-1 Statistical table. All tests were performed on normally distributed data. Shown are (***a–l***) Tukey’s multiple comparisons test for significant ANOVAs and (***m***, ***n***) unpaired *t* test with Welch’s correction. Download Table 2-1, DOCX file.

10.1523/ENEURO.0406-22.2023.f3-1 Extended Data Figure 3-1 MK-801 during the “still” period affects the power spectrum (PS) by increasing higher frequencies in the periodic component and lower frequencies in the aperiodic. PSs (***A***), periodic component (***B***), aperiodic component (***C***) in the primary auditory (left; *N* = 5), parietal (center; *N* = 11), and frontal cortex (right; *N* = 8) before dosing (grey) and under the effects of MK-801 (red). Solid lines indicate the average and shaded areas describe the SEM. Top horizontal bars indicate clusters identified by paired CBP analysis (lighter shade *p* < 0.01; darker shade *p* < 0.05). Download Figure 3-1, EPS file.

To compare each feature between the vehicle and the compounds, a two-way repeated-measures ANOVA was performed. To increase the robustness of the statistical analysis, experiments 1 and 2 were merged by including the experiment number and the age at the time of recording as regressors. The ANOVAs were performed on the resulting residuals. Normality was assessed with the One-sample Kolmogorov–Smirnov test (MATLAB’s *kstest()* function), and, in case of rejection, data were transformed to fit a normal distribution as described by (van Albada and Robinson, 2007). Only animals with high-quality sessions in all regions under all conditions were included, giving a total *N* = 5. In the case of the modal frequency parameter, no frequency can be measured when there is no amplitude. This results in very few or no paired modal frequency measures for each band. Therefore, no peak frequencies were included in the ANOVA analysis. In cases where interaction was confirmed, *post hoc* Tukey’s analysis was performed. In the cases where no interaction was detected, but there were changes in the treatment, a one-way repeated-measures ANOVA and posterior *post hoc* Tukey’s analysis were performed.

For the MK-801 condition, one animal’s exposure could not be determined because it was below the detection limit of the technique. Outliers in the exposure values were identified through the *ROUT* method (Q = 5%) in GraphPad Prism. One value of exposure in the case of MK-801 and one in the case of diazepam were determined outliers and excluded from further analysis (Extended Data Fig. 7-1). As in the case of the ANOVA, experiments 1 and 2 were merged by including the experiment number and the age at the time of recording as regressors. The Spearman correlation coefficient between exposures and EEG parameters was computed. For the amplitude and the AUC, the correlation was calculated on the difference between the resulting residuals of the compound of interest (diazepam or MK-801) and the vehicle sessions. For the modal frequencies, since in some cases the compounds induce the appearance of an oscillatory band that is absent under the vehicle (e.g., HFO generated by MK-801), the compound residual (not normalized to vehicle) is used. To control for multiple correlations, a permutation analysis was performed to determine the statistical significance. For each parameter of interest, the Spearman correlation to exposure was calculated, then the data were permuted (1000 permutations) and for each permutation, a new Spearman coefficient was calculated. The resulting *p*-value reflects the number of times the correlation coefficient was stronger than the observed correlation.

### Code accessibility

The code needed to reproduce the analysis described in this paper can be accessed from GitHub (https://github.com/SystemNeuroCIMA/eNeuroGonzalezBurgos) on reasonable request to the authors.

## Results

The PSs reveal unique brain region-specific pharmacodynamic qEEG signatures depending on the compound administered. As expected, after the administration of diazepam, PS analysis reveals a robust increase at ∼10–20 Hz (β band oscillatory activity) in all regions studied (Fig. 2*A*; Table 2). Additionally, there is a wideband increase of slow frequencies (∼0.1–10 Hz) and a robust reduction in the high-frequency range across all regions studied (∼60–150 Hz; Table 2). The decreasing tendency of the power of the frequency spectra evidences the need to further analyze the PS signal by decomposing it into periodic and aperiodic components. Analysis of the periodic component confirms the robust ∼10- to 20-Hz (β band) increase induced by diazepam while also clarifying an increase in the ∼20- to 60-Hz (γ band) oscillations (Fig. 2*B*; Table 2). On the other hand, analysis of the aperiodic component identified the steepening of the 1/f slope induced by diazepam (Fig. 2*C*; Table 2).

**Table 2:** Summary of results

		Diazepam			MK-801		
		Auditory	Parietal	Frontal	Auditory	Parietal	Frontal
Power Spectrum		↑ Beta oscillations (∼10–20 Hz) ↑ Slow frequencies (∼0.1–10 Hz) ↓ High frequencies (∼60–150 Hz)			↓ Beta oscillations (∼10–20 Hz) ↑ Gamma oscillations (∼30–100 Hz) Appearance of HFO (∼150 Hz)		
Aperiodic		↑↑↑ Slope in all regions ↑↑↑ Offset in all regions ↑↑ Knee in all regions			↑ Slope in all regions		
					∼ Offset	↑ Offset	∼ Offset
					↑↑↑ Knee in all regions		
Periodic	Theta	↓ Amp ↓ AUC	↓↓↓ Amp ↓↓ AUC	∼ Amp ∼ AUC	∼		
	Beta	↑ Amp in all regions ↑ AUC in all regions			↓ Amp in all regions ↓ AUC in all regions		
	Low-gamma	∼			∼		
	Mid-gamma	↑↑ Amp ∼ AUC	∼ Amp ∼ AUC	∼ Amp ∼ AUC	∼		
	High-gamma	Auditory < Parietal < Frontal					
	HFO	∼			↑ Amp ↑ AUC	↑↑ Amp ↑↑ AUC	↑↑↑ Amp ↑↑↑ AUC

For each compound, changes within each region are described as ∼ when no change occurs, ↑ when there is an increase, and ↓ when there is a decrease. The number of arrows indicates the intensity of the change. Details of the statistical analyses can be found in Extended Data Table 2-1.

In addition, we found that MK-801 leads to the expected decrease in ∼10- to 20-Hz (β band) oscillations, an increase in ∼30- to 100-Hz (γ band) oscillations, and an induction of ∼150-Hz activity (HFO; Fig. 3*A*; Table 2). However, the magnitude of the changes in the β range is dependent on the brain region over which they are recorded, being less pronounced in the primary auditory cortex and most evident in the frontal cortex. Furthermore, the HFO are most clearly observed in the frontal cortex, less so in the parietal cortex, and not observed over the primary auditory cortex, as shown in Figure 3*A* (Table 2). The region-specific effects of the NMDAR antagonist suggest a neuronal circuit-specific dynamic shift that consequently leads to distinct neurophysiological readouts. Similarly to what is observed in diazepam, the decomposition of the PS under MK-801 evidenced changes in the periodic and aperiodic components. Analysis of periodic components not only confirms the expected oscillatory changes but it elucidates the appearance of HFO also in the primary auditory cortex (Fig. 3*B*; Table 2), which is not discernable in the classical PS. In addition, there is a shift in the frequency at which the “knee” appears (Fig. 3*C*; Table 2). These results reinforce the need to decompose PS signals into periodic and aperiodic components to evaluate pharmacologically induced changes adequately.

### Diazepam and MK-801 induce significant alterations in the aperiodic component

**Figure 4.**
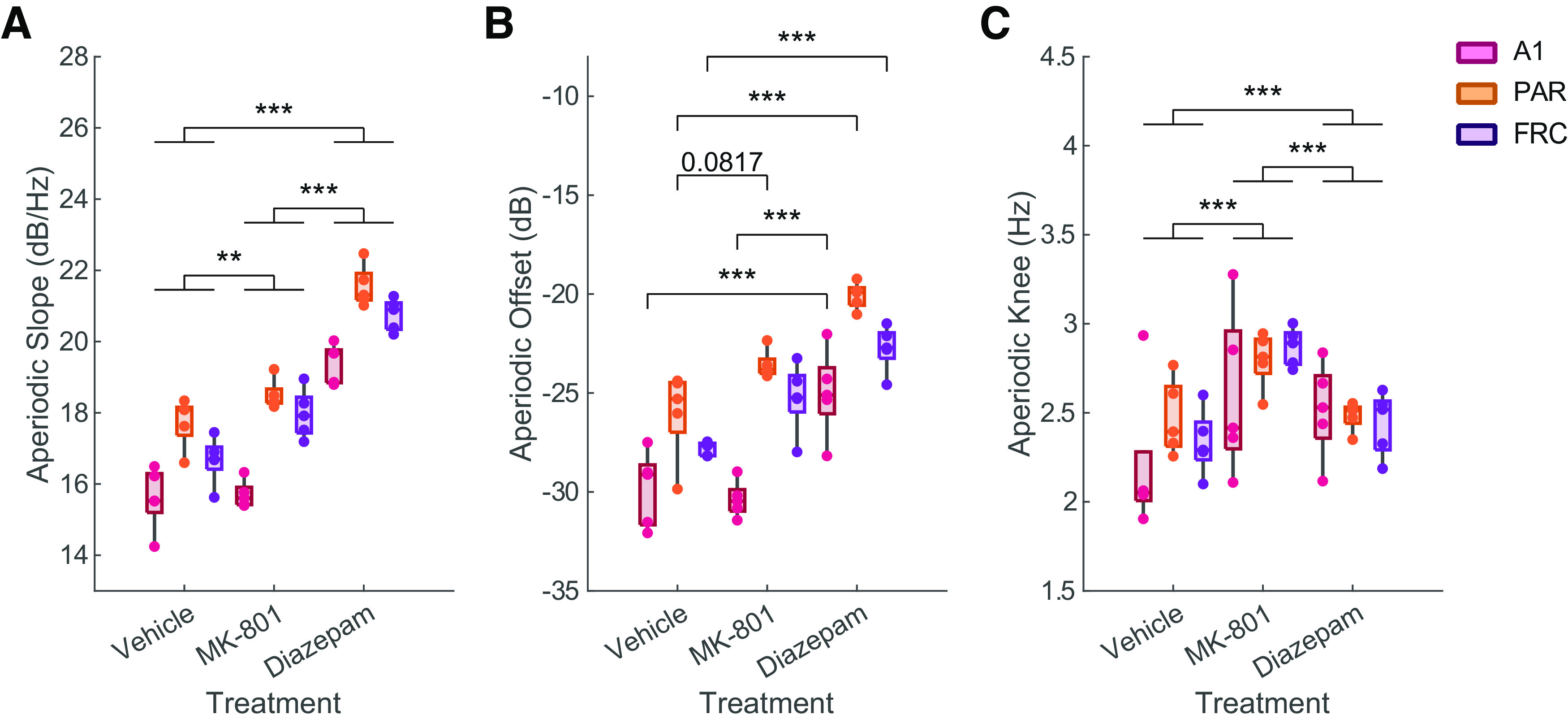
Quantitative changes induced by diazepam and MK-801 on the aperiodic slope (***A***), offset (***B***), and “knee” (***C***) in the primary auditory (A1), parietal (PAR), and frontal (FRC) cortices (*N* = 5). Tukey’s *post hoc* tests *p* < 0.1 are shown. *p < 0.05, **p < 0.01, ***p < 0.001.

Once the expected compound-specific EEG signatures were confirmed, we proceeded to quantify the magnitude of the changes described. Starting with the aperiodic component of the PS signal, the aperiodic slope is distinctly different depending on the compound administered (*F*_(2,8)_ = 90.61, *p* < 0.001; Fig. 4*A*) or the brain region studied (*F*_(2,8)_ = 57.15, *p* < 0.001; Fig. 4*A*), although there is no interaction between the two factors (*F*_(4,16)_ = 1.75, *p* = 0.189; Fig. 4*A*). Specifically, diazepam robustly increases the slope as compared with vehicle (*p* < 0.001; Extended Data Table 2-1*a*), while MK-801 also increases it although less dramatically (*p* = 0.004; Extended Data Table 2-1*a*). Complementary to the changes in slope, the aperiodic offset is also distinct depending on the interaction between the compound administered and the region studied (*F*_(4,16)_ = 3.191, *p* = 0.042; Fig. 4*B*). *Post hoc* analysis reveals an increase in the aperiodic offset induced by diazepam in all three regions studied as compared with the vehicle (*p* < 0.001; Extended Data Table 2-1*b*), and only in the auditory cortex when compared with MK-801 (*p* < 0.001; Extended Data Table 2-1*b*). To conclude the analysis of the aperiodic component, the frequency of the “knee” was also highly informative, being distinctly different depending on the compound administered (*F*_(2,8)_ = 12.98, *p* = 0.003), although not on the region studied (*F*_(2,8)_ = 0.53, *p* = 0.607; Fig. 4*C*) or the interaction between the compound and region (*F*_(4,16)_ = 1.13, *p* = 0.377). The frequency of the “knee” increases in the case of both the diazepam and the MK-801 (Fig. 4*C*; *p* < 0.001; Extended Data Table 2-1*c*), being more pronounced in the case of MK-801 (*p* < 0.001; Extended Data Table 2-1*c*). All these results show that diazepam at 2 mg/kg and MK-801 at 0.075 mg/kg lead to dramatic changes in the aperiodic component (Table 2).

**Figure 5.**
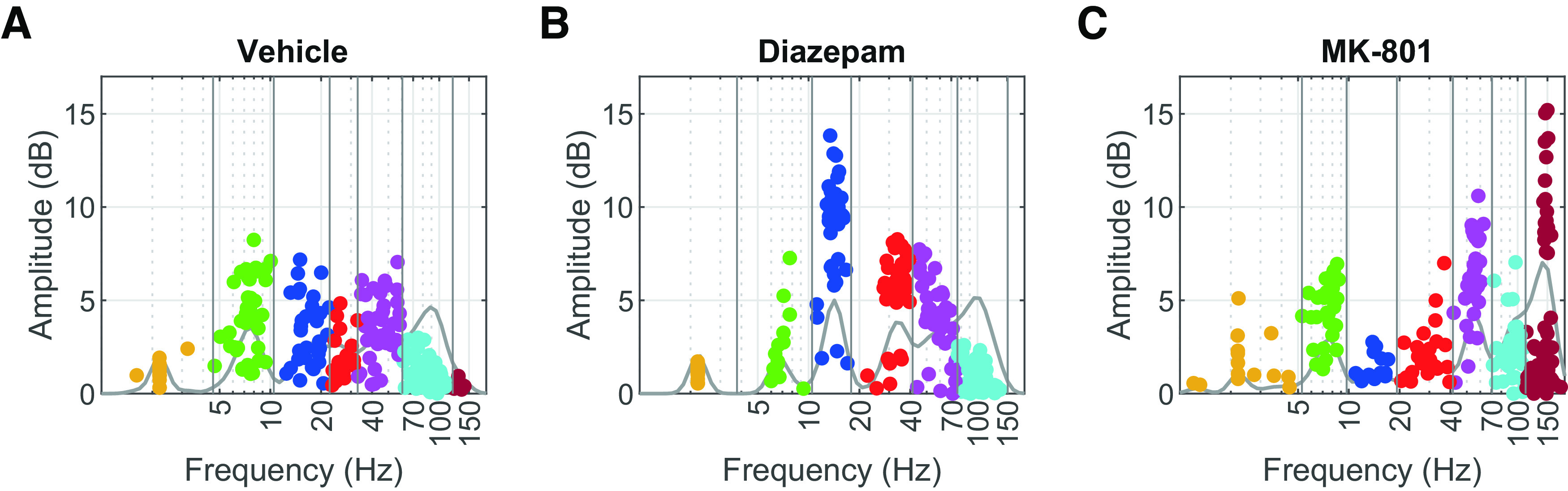
Distribution of Gaussian functions fitted to the periodic component determine the compound and state-specific limits of δ (yellow), θ (green), β (blue), low-γ (red), mid-γ (magenta), high-γ (cyan), and HFO (burgundy) frequency bands (***A***) under the vehicle, (***B***) under diazepam, and (***C***) under MK-801. Dots represent the totality of Gaussian functions among all animals and all brain regions. Superimposed traces indicate kernel-density estimates, and horizontal lines indicate frequency band limits.

### Diazepam and MK-801 alter the periodic component

To quantify the changes in the periodic component appropriately, we decomposed it into a series of Gaussian kernels. We considered their frequency distribution to determine the frequencies where oscillatory modes appear. We defined them in terms of three conditions: postdosing sessions for vehicle, diazepam, and MK-801. We found a total of seven clearly defined frequency bands (Fig. 5), each condition with unique limits to these frequency bands (Table 1).

**Figure 6.**
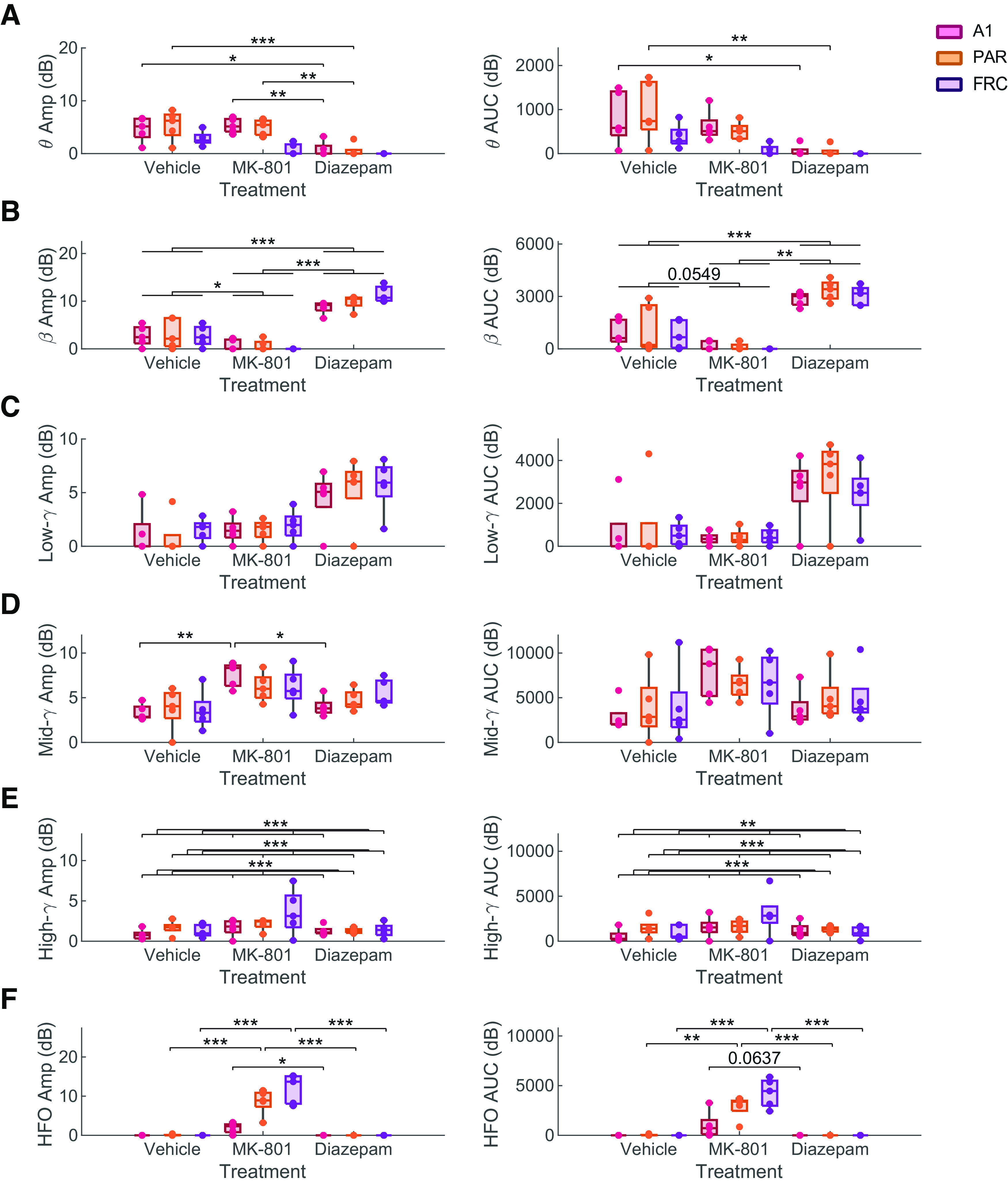
Quantification of peak amplitude (left) and AUC (right) in the (***A***) θ (VEH: 5.3–9.7; DZP: 4.6–10.5; MK: 4.0–9.0 Hz), (***B***) β (VEH: 9.7–19.3; DZP: 10.5–22.5; MK: 9.0–19.3 Hz), (***C***) low-γ (VEH: 19.3–41.3; DZP: 22.5–32.9; MK: 19.3–32.9 Hz), (***D***) mid-γ (VEH: 41.3–70.5; DZP: 32.9–60.5; MK: 32.9–60.5 Hz), (***E***) high-γ (VEH: 70.5–111.4; DZP: 60.5–120.2; MK: 60.5-111.4 Hz), and (***F***) high-frequency (HFO; VEH: >111.4; DZP: >120.2; MK: >111.4 Hz) oscillatory bands in the primary auditory (A1), parietal (PAR), and frontal (FRC) cortices (*N* = 5). Tukey’s *post hoc* tests *p* < 0.1 are shown. *p < 0.05, **p < 0.01, ***p < 0.001.

PS decomposition and posterior quantification of the bands confirmed previously described changes and revealed additional findings (Table 2). Expected oscillatory changes induced by diazepam include a robust increase in the β band amplitude (Fig. 6*B*; *F*_(2,8)_ = 16.79, *p* = 0.001; *p* < 0.001; Extended Data Table 2-1*f*) and AUC (Fig. 6*B*; *F*_(2,8)_ = 13.67, *p* = 0.003; *p* < 0.001; Extended Data Table 2-1*g*). An additional finding is the changes in the θ oscillatory band amplitude (Fig. 6*A*; *F*_(4,16)_ = 14.59, *p* < 0.001; Extended Data Table 2-1*d*) and AUC (Fig. 6*A*; *F*_(4,16)_ = 4.91, *p* = 0.009; Extended Data Table 2-1*e*), which depend on the interaction between the brain region and the compound administered. Diazepam decreases θ oscillations in the auditory (amplitude *p* = 0.014; AUC *p* = 0.044; Extended Data Table 2-1*e*) and parietal cortices (amplitude *p* = 0.001; AUC *p* = 0.006; Extended Data Table 2-1*e*), but not in the frontal cortex (Fig. 6*A*).

On the other hand, predicted MK-801-induced changes include an increase in mid-γ amplitude (Fig. 6*D*; *F*_(4,16)_ = 4.15, *p* = 0.017; Extended Data Table 2-1*h*), and HFO amplitude (Fig. 6*F*; *F*_(4,16)_ = 7.38, *p* = 0.001; Extended Data Table 2-1*k*) and AUC (Fig. 6*F*; *F*_(4,16)_ = 5.05, *p* = 0.008; Extended Data Table 2-1*i*). Interestingly, both mid-γ and HFO changes induced by MK-801 are region-dependent. MK-801-induced HFO changes are more pronounced in the frontal (Fig. 6*F*; *p* < 0.001; Extended Data Table 2-1*k*,*I*) and parietal cortices (Fig. 6*F*; amplitude *p* < 0.001; AUC *p* = 0.002; Extended Data Table 2-1*k*,*I*). In the auditory cortex, the HFO are larger than in diazepam in amplitude (Fig. 6*F*; *p* = 0.016; Extended Data Table 2-1*k*) and AUC (Fig. 6*F*; *p* = 0.064; Extended Data Table 2-1*I*). Remarkably, although there is a clear increase in mid-γ amplitude in the auditory cortex (Fig. 6*D*; *F*_(4,16)_, *p* =0.017; *p* = 0.001; Extended Data Table 2-1*h*), this increase is not found in the frontal (*p* = 0.163; Extended Data Table 2-1*h*) or parietal cortices (*p* = 0.220; Extended Data Table 2-1*h*). These data further support the region specificity of the changes to the EEG signal induced by the compounds.

Highlighting the region specificity of the bands, it is worth noting that high-γ amplitude (Fig. 6*E*; *F*_(2,8)_ = 2.68; *p* = 0.045; Extended Data Table 2-1*i*) and AUC (*F*_(2,8)_ = 5,12; *p* = 0.037; Extended Data Table 2-1*i*) are region-dependent, being the largest in the frontal (*p* = 0.001; Extended Data Table 2-1*i*) and the smallest in the auditory cortex (*p* < 0.001; Extended Data Table 2-1*i*). These results emphasize the need to analyze each region individually.

### Compound plasma exposure correlates with aperiodic and periodic parameters of the PS in a region-specific manner

Careful observation of the data revealed between-subject variability of qEEG measures. Consequently, we set out to determine whether drug exposure could account for such variability. We employed bioanalytical analysis to capture the variability in drug exposure and investigate its impact on pharmacodynamic measures. Of note, animals in experiment 1 present higher plasma concentrations of MK-801 (*t*_(8.411)_ = 2.382, *p* = 0.0430; Extended Data Table 2-1*m*) and diazepam (*t*_(8.041)_ = 4.699, *p* = 0.0015; Extended Data Table 2-1*n*) compared with experiment 2. The measured exposures help us control for variability introduced by potential differences in formulation, age of the animals tested, and other unknown factors. Moreover, single-dose experiments offer a limited exposure range, which purposely challenges the sensitivity and value of qEEG readouts.

**Figure 7.**
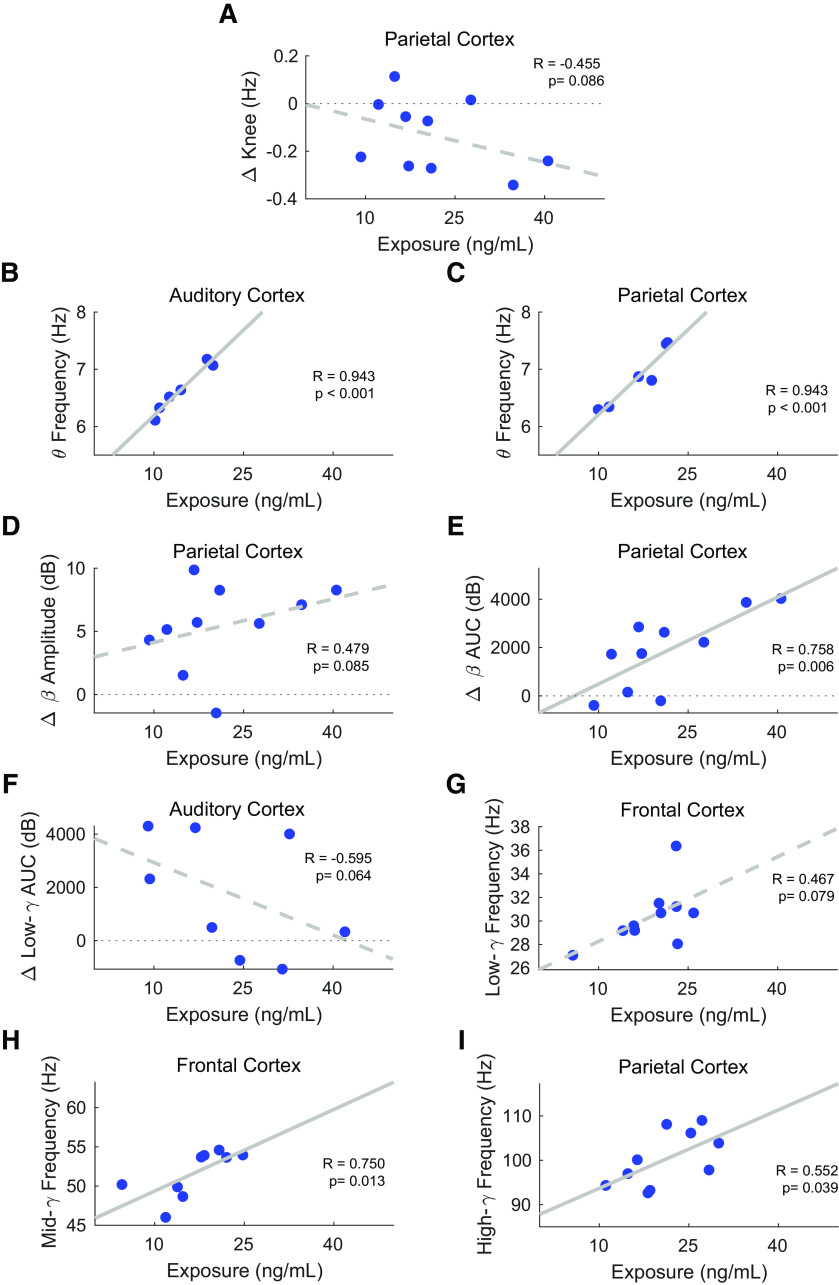
Diazepam plasma concentration correlates to (***A***) aperiodic knee in parietal cortex, (***B***) θ modal frequency in auditory cortex, (***C***) θ modal frequency in parietal cortex, (***D***) β amplitude in parietal cortex, (***E***) β AUC in parietal cortex, (***F***) low-γ AUC in auditory cortex, (***G***) low-γ modal frequency in frontal cortex, (***H***) mid-γ modal frequency in frontal cortex, and (***I***) high-γ modal frequency in frontal cortex. Two-tail Spearman correlation coefficients and *p*-values are shown. Least-squares line is drawn dotted when *p* < 0.1 or solid when *p* < 0.05 (*N*_permutations_ = 1000). Of note, diazepam exposure is larger and animals are younger in experiment 1 than in experiment 2 (Extended Data Fig. 7-1), so experiment number and age are used as regressors in the analysis.

In the case of diazepam, concerning the aperiodic 1/f component, the knee frequency tends to correlate negatively to the exposure in the parietal cortex (Table 3; Fig. 7*A*; *R* = −0.455). Although not significant, this negative tendency is seen in other electrodes (Table 3). No correlation could be established for the aperiodic slope and offset (Table 3). Concerning the oscillatory component, the general trend is for modal frequencies to increase with exposure and for amplitudes and AUC to correlate with an increase in β band oscillations. As such, θ modal frequency in the auditory (Table 3; Fig. 7*B*; *R* = 0.943) and parietal (Table 3; Fig. 7*C*; *R* = 0.943) cortices positively correlates to exposure. In other words, the higher the plasma concentration of diazepam, the closer to the β band range the θ band oscillates. Also positively correlating to exposure is the β band amplitude (Table 3; Fig. 7*D*; *R* = 0.479) and AUC (Table 3; Fig. 7*E*; *R* = 0.758) in the parietal cortex. The positive correlation between β oscillations and diazepam exposure is extremely relevant since this is the most salient PS feature induced by the compound. In contrast, low-γ AUC tends to correlate negatively to exposure in the auditory cortex (Table 3; Fig. 7*F*; *R* = −0.595). However, low-γ modal frequency tends to correlate positively with exposure (Table 3; Fig. 7*G*; *R* = 0.467) in the frontal cortex. The increase in modal frequencies in the γ range also occurs for mid-γ in the frontal (Table 3; Fig. 7*H*; *R* = 0.750) and for high-γ in the parietal cortex (Table 3; Fig. 7*I*; *R* = 0.552).

**Table 3:** Two-tail Spearman correlation coefficients and *p*-values (*N*_permutations_ = 1000) for each of the aperiodic and periodic parameters extracted for diazepam and MK-801 in the primary auditory, parietal, and frontal cortices

		Diazepam						MK-801					
		Auditory cortex		Parietal cortex		Frontal cortex		Auditory cortex		Parietal cortex		Frontal cortex	
		Rho	*p*-value	Rho	*p*-value	Rho	*p*-value	Rho	*p*-value	Rho	*p*-value	Rho	*p*-value
Aperiodic slope		0.238	0.274	0.370	0.134	0.333	0.181	−0.300	0.249	0.685	0.016*	−0.143	0.358
Aperiodic offset		0.310	0.216	0.164	0.321	0.283	0.221	−0.300	0.274	0.406	0.123	−0.286	0.245
Aperiodic “knee”		−0.238	0.269	−0.455	0.086^+^	−0.383	0.150	−0.300	0.265	0.285	0.195	−0.500	0.120
θ	Amplitude	−0.286	0.217	−0.176	0.306	0.417	0.137	0.100	0.371	−0.285	0.190	0.357	0.207
	AUC	−0.357	0.204	−0.212	0.278	0.317	0.188	0.300	0.250	−0.745	0.007**	0.071	0.419
	Frequency	0.943	<0.001***	0.943	<0.001***			−0.333	0.188	0.073	0.427		
β	Amplitude	0.167	0.331	0.479	0.085^+^	0.117	0.370	0.100	0.395	−0.115	0.382	0.321	0.234
	AUC	0.381	0.164	0.758	0.006*	0.333	0.163	0.600	0.094^+^	0.212	0.252	0.429	0.147
	Frequency	−0.077	0.406	0.073	0.4	0.018	0.454			−0.200	0.343		
Low-γ	Amplitude	−0.452	0.108	−0.382	0.123	−0.283	0.224	−0.900	0.005**	0.394	0.123	−0.357	0.200
	AUC	−0.595	0.064^+^	−0.139	0.352	−0.050	0.419	−1.000	<0.001***	0.067	0.408	−0.357	0.171
	Frequency	0.103	0.366	−0.042	0.454	0.467	0.079^+^	0.257	0.265	0.143	0.378	−0.786	0.005**
Mid-γ	Amplitude	0.429	0.126	0.006	0.459	0.167	0.320	0.800	0.048*	0.200	0.274	0.143	0.343
	AUC	0.452	0.125	0.236	0.264	0.133	0.339	0.900	0.008**	0.188	0.290	−0.036	0.457
	Frequency	−0.018	0.445	0.082	0.419	0.750	0.013*	0.143	0.337	0.309	0.182	0.286	0.225
High-γ	Amplitude	−0.024	0.451	−0.236	0.254	−0.033	0.458	0.000	0.480	−0.042	0.448	−0.286	0.255
	AUC	0.167	0.308	−0.224	0.237	0.083	0.400	−0.100	0.388	−0.127	0.311	0.071	0.432
	Frequency	0.476	0.104	0.552	0.039*	−0.176	0.304	0.543	0.116	0.617	0.040*	0.524	0.085^+^
HFO	Amplitude			−0.079	0.410	0.276	0.233	0.300	0.249	−0.273	0.203	0.036	0.415
	AUC			−0.091	0.387	0.276	0.217	0.300	0.231	−0.394	0.128	0.036	0.456
	Frequency							−0.371	0.217	0.445	0.076^+^	0.786	0.007**

+*p* < 0.1, *p < 0.05, **p < 0.01, ***p < 0.001.

10.1523/ENEURO.0406-22.2023.f7-1 Extended Data Figure 7-1 Relationship between the age of the animals and exposure to (***A***) diazepam and (***B***) MK-801 (ng/ml). Experiment 1 animals (orange) show larger exposures than experiment 2 animals (purple). Data points identified as outliers by the ROUT method (Q = 5%) are identified with an asterisk. Download Figure 7-1, EPS file.

**Figure 8.**
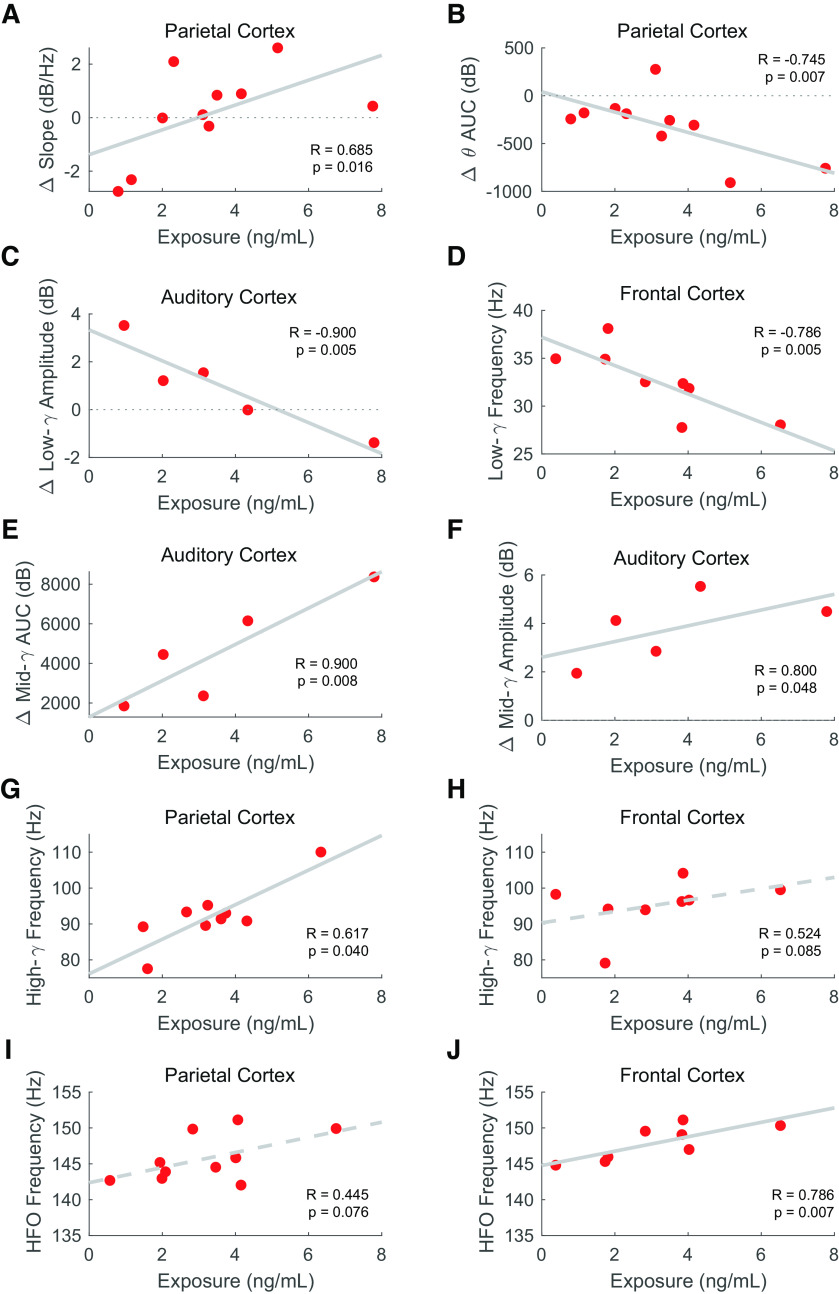
MK-801 plasma concentration correlates to (***A***) aperiodic slope in parietal cortex, (***B***) θ AUC in parietal cortex, (***C***) low-γ amplitude in auditory cortex, (***D***) low-γ modal frequency in frontal cortex, (***E***) mid-γ AUC in auditory cortex, (***F***) mid-γ amplitude in auditory cortex, (***G***) high-γ modal frequency in the parietal cortex, (***H***) high-γ modal frequency in the frontal cortex, (***I***) high-frequency oscillations (HFO) modal frequency in parietal cortex, and (***J***) HFO modal frequency in frontal cortex. Two-tail Spearman correlation coefficients and *p*-values are shown. Least-squares line is drawn dotted when *p* < 0.1 or solid when *p* < 0.05 (*N*_permutations_ = 1000). Of note, MK-801 exposure is larger and animals are younger in experiment 1 than in experiment 2 (Extended Data Fig. 7-1), so experiment number and age are used as regressors in the analysis.

Similarly, correlations between relevant EEG parameters and exposure can be established for MK-801. Specifically, the aperiodic slope (Table 3; Fig. 8*A*; *R* = 0.685) positively correlates with exposure in the parietal region. No such correlation can be established in the primary auditory and frontal cortices. Regarding the oscillatory changes, θ AUC negatively correlated to MK-801 exposure in the parietal cortex (Table 3; Fig. 8*B*; *R* = −0.745). Apart from θ, the γ frequency range proved to be very informative. The amplitude (Table 3; Fig. 8*C*; *R* = −0.900) and AUC (Table 3; *R* = −1.000) of the low-γ peak negatively correlates to exposure in the auditory cortex, while the modal frequency of the peak negatively correlates in the frontal cortex (Table 3; Fig. 8*D*; *R* = −0.786). The amplitude (Table 3; Fig. 8*E*; *R* = 0.800) and AUC of mid-γ (Table 3; Fig. 8*F*; *R* = 0.900) positively correlate with exposure in the auditory cortex, while the modal frequency of high-γ positively correlates to exposure in parietal (Table 3; Fig. 8*G*; *R* = 0.617) and frontal cortex (Table 3; Fig. 8*H*; *R* = 0.524). Relevantly, HFO parameters also correlate to MK-801 exposure. Specifically, the modal frequency of HFO positively correlates to exposure in the parietal (Table 3; Fig. 8*I*; *R* = 0.445) and frontal cortex (Table 3; Fig. 8*J*; *R* = 0.786).

## Discussion

This work aimed to investigate which qEEG features best describe EIB modulation. Here, we analyzed EEG activity of freely moving rats under EIB pharmacological modulation and detected novel qEEG features sensitive to relatively small changes in plasma exposures. This is concluded from the decomposition of the PS profiles of diazepam and MK801 into their periodic and aperiodic components. To our knowledge, this is the first report to use PS decomposition to discover exposure-dependent signatures of MK801 and diazepam across brain regions and EEG components.

Previous research shows that NMDAR antagonists and GABAAR modulators induce variations to the qEEG signal that reflect effects on EIB. Specifically, NMDAR antagonism has been shown to increase γ and HFO in humans (McNally and McCarley, 2016) as well as in rodents (Flores-Barrera et al., 2020). Also conserved across species are some effects of GABAAR modulation in the form of robust increases in β and alterations in γ oscillatory activity in humans (Jobert and Wilson, 2015; Premoli et al., 2017), rodents (Christian et al., 2015), and monkeys (Berro et al., 2021). Our results build on these validated translational biomarkers of brain circuit activity and extend these findings by identifying qEEG features that are sensitive to small changes in plasma exposures. First, we show that PS signatures are not only compound-specific, but they present region-specific phenotypes. Second, by decomposing the PS into their periodic and aperiodic components, we show distinct effects that were not apparent through conventional analysis. Third, we illustrate how to determine the limits of the oscillatory bands, thus allowing us to quantify their variation in a natural and unbiased manner. Notably, our analyses revealed that parameters of the qEEG correlate with plasma concentrations, demonstrating the sensitivity of the PS to reflect exposure.

### PS signatures show not only compound but also region-specific phenotypes

In this work, we show how brain region-specific activity patterns emerge from systemic pharmaco-modulation of EIB. Differences across brain regions are observed in the aperiodic and periodic components extracted through the decomposition of the PS. The correlations between EEG features and plasma exposure are also dependent on the brain region where the signal originates.

**Figure 9.**
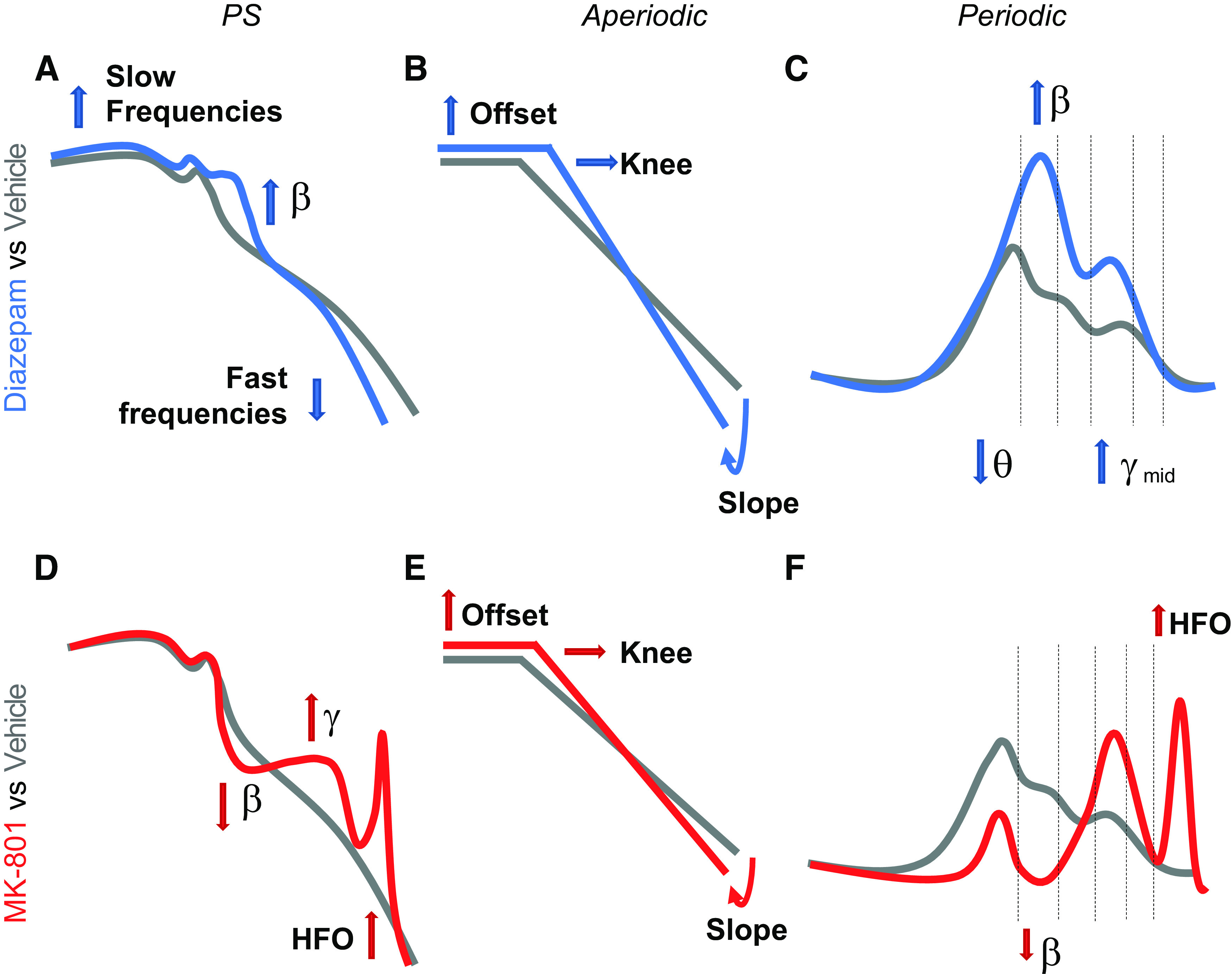
Graphical summary of results. ***A***, Power spectrum (PS) after administration of diazepam evidences an increase in slow frequencies, an increase in β band oscillations, and a decrease in fast frequencies. ***B***, Aperiodic component of the PS evidences an increase in slope accompanied by an increase in offset and “knee” frequency. ***C***, Periodic analysis confirms an increase in β band oscillations, while evidencing a decrease in θ and an increase in mid-γ band oscillations. ***D***, MK-801 leads to an increase in γ band oscillations, a decrease in β band oscillations, and the appearance of high-frequency oscillations (HFOs) in the PS. ***E***, Aperiodic component of the PS evidences an increase in slope accompanied by an increase in offset and “knee” frequency. ***F***, Periodic decomposition of the PS confirms the decrease in β activity and the appearance of HFO.

PS analysis of NMDAR and GABAAR modulation recreates previously published findings. Specifically, NMDAR antagonism increases γ band and HFO power (Fig. 9*D*), a systematic finding in clinical and preclinical studies (Bianciardi and Uhlhaas, 2021). Also for GABAAR modulation, our data indicate an increase in β band oscillatory activity (Fig. 9*A*), confirming results from previous clinical and preclinical studies (Ahmad, et al., 2022). These are two conventional biomarkers of EIB. Apart from corroborating their relevance, we have found that the magnitude is brain region and, in some cases, concentration dependent.

Previous work reported the aperiodic component to be brain region-specific under pharmacological modulation (Valencia et al., 2012) or specific behavioral tasks (He et al., 2010). Although the reasons for the region specificity are unknown, a simple explanation is that the signal detected by the recording electrode depends on its distance to the source of the oscillations. For example, HFO have been proposed to originate in the olfactory bulb (Hunt et al., 2018). An anterior origin of HFO may explain why when administering MK-801 the HFO are more prominent in the frontal electrode while diminishing as the electrodes are placed posteriorly first in the parietal cortex and then in the auditory cortex. A more complex explanation for region-specific electrophysiological readouts is that the regions studied have distinct neuronal circuitry that conditions the pharmacological modulation. The effect of modulating a receptor (i.e., MK-801 modulating NMDAR) will depend on the population density and location of cells that express the target receptor, the density of the receptors themselves, and the connectivity of the modulated cells within the nuclei and with other regions. In this study, three regions with distinct functionalities have been studied: the primary auditory cortex is a primary sensory cortex, the parietal cortex mediates sensorimotor processing, and the frontal cortex is involved in higher-order cognitive processing. These distinguishable functionalities are a reflection of the distinct anatomic composition and neuronal connectivity of these regions. The region-specific effects are reflected as precise neurophysiological readouts. Our findings emphasize the complex and varied effects of brain-wide neurotransmitter system modulators, which act on a multitude of circuits differentially affecting regional EEG.

### The aperiodic component of PS is sensitive to EIB modulations

Apart from the region-specificity, we found that by dissecting the EEG PS into periodic and aperiodic components, we regain information that would otherwise be lost with more canonical approaches to EEG analysis. For example, diazepam-driven increases in low-γ oscillations are masked by changes in the periodic slope in the PS analysis (Fig. 9*C*). This is relevant given the supposed role of γ oscillations on higher-order cognitive processes, indicating that GABAAR modulation may also interfere with these processes. On the other hand, classical PS representations under MK-801 did not reveal robust effects in the slope of the PS. However, through the decomposition, we found that MK-801 tended to steepen the 1/f slope. Considering that MK-801 is often employed as a pharmacological model of schizophrenia, our results neatly align with reports of steeping of the 1/f component in schizophrenia patients (Peterson et al., 2023). Other studies have reported a flattening of the curve under ketamine, a different NMDAR antagonist (Muthukumaraswamy and Liley, 2018). However, the decomposition method of the PS used in that study divided the spectra into two ranges: before and after the knee. Electrophysiological signals may require a different fitting function depending on species, physiological condition, electrode type, electrode placement, etc. Here, we explored a family of decreasing functions and identified that our data were best fitted by using a Lorentzian function. By doing so, we were able to parametrize two spectral ranges separated by a knee at a frequency that is determined in a nonbiased manner (Donoghue et al., 2020; Extended Data Fig. 2-1). Regardless of the decomposition method used, our results further evidence the need to dissect the PS estimate to assess electrophysiological changes properly.

Speculating on the cause of the changes to the 1/f component is challenging, considering that little is known about its driving force. It has a physiological relevance as it is dependent on chronological age (Voytek et al., 2015; Waschke et al., 2017; Dave et al., 2018; Bódizs et al., 2021; Cellier et al., 2021), task (Podvalny et al., 2015; Ouyang et al., 2020; Waschke et al., 2021), and attention (He, 2014; Donoghue et al., 2020; Thuwal et al., 2021; Waschke et al., 2021). This component has also been described to be altered in neurodevelopmental (Neklyudova et al., 2022), neuropsychiatric (Peterson et al., 2023), and neurodegenerative disorders (Kim et al., 2022). Furthermore, this component is modulated by pharmacology (Valencia et al., 2012; Muthukumaraswamy and Liley, 2018). Several mechanisms for generating the aperiodic component have been proposed, such as the self-organized criticality theory (Beggs, 2008), the frequency dependence of current propagation in biological tissues (Bédard and Destexhe, 2009), or the mixture of damped neuronal oscillators having a distribution of relaxation states (Muthukumaraswamy and Liley, 2018). Another theory proposes that the 1/f activity can be attributable to the low-pass filtering property of dendrites, which attenuate faster signals more than slow ones (Lindén et al., 2010; Buzsáki et al., 2012). This would explain why slower frequencies present higher power than faster frequencies. Moreover, the low-pass filtering action of each cell depends not only on the dendrites’ length but also on each cell’s input electrical resistance. These characteristics are susceptible to physiological states. Therefore, under this assumption, the nature and state of the cells that integrate the circuit would be key to determining the 1/f component. Moreover, excitatory or inhibitory disturbances that alter the conductance properties of the cells that integrate the circuits would lead to changes in the 1/f component that can be registered with the qEEG. Considering this, it would make sense for receptor-specific pharmacomodulation to alter the conductance properties of the cells that integrate the circuit and cause EI disruption reflected as a change in the 1/f slope. Thus, pharmacologically-induced changes in the periodic oscillatory activity would reflect differential recruitment and synchronization of neuronal populations at specific frequencies, while aperiodic changes would reflect circuit-wide conductance changes. Nevertheless, our results confirm previous studies that described the 1/f slope as a marker of EIB (Ahmad, et al., 2022).

### Unbiased decomposition of periodic component of PS under EIB modulation

In line with the aforementioned changes in EIB induced by pharmacological compounds, we found shifts in the frequency of the peaks of the oscillatory bands. It is, therefore, essential to determine such peaks for every condition investigated. One can conclude that when exploring PS estimates, the changes in the oscillatory bands may not be due exclusively to changes in power but also to changes in the frequency at which they appear. Fixating on predefined standard limits of oscillatory bands risks misinterpreting changes in power with changes in frequency. For example, a shift of the β band oscillation toward slower frequencies may result in an overestimation of the α or θ range. The unspecific biased definition of the frequency bands may explain why there are incongruencies regarding the correlation between oscillatory activity and function. For example, α oscillatory activity has been simultaneously described to correlate (Ergenoglu et al., 2004) and not correlate to cognitive performance (Ouyang et al., 2020). We believe that the proposed decomposition of the periodic component into Gaussians circumvents this confounding situation. We suggest defining the oscillatory bands and their corresponding frequency limits by how they are presented under each specific experimental condition. To do so, we suggest defining the limits of the oscillatory bands by estimating the density of Gaussian oscillations in the periodic component along the frequency spectrum.

Additionally, we observed that pharmacological modulation causes shifts in the PS power distribution and not necessarily adjustments in the overall total power. We have shown that, apart from changes to the 1/f component, disruptions to the EIB can be manifested through oscillatory changes in two ways: (1) the power within an oscillatory band is maintained but there is a shift toward a preferred frequency, manifested as a constant AUC but an increase in amplitude; or (2) the power shifts toward an oscillatory band in detriment of other bands, manifested as simultaneous increases or decreases in both AUC and amplitude. We have seen that quantifying the parameters of the extracted oscillatory component is key in analyzing the complex pharmacologically-driven modulations to the EIB, which act on intricate circuit dynamics.

### Features of the periodic and aperiodic components are sensitive to plasma concentrations

This study shows the great power in connecting pharmacodynamics parameters to measured drug exposures. The doses and postdosing recording times were chosen to achieve exposures that maximize physiological changes with minimal behavioral confounds. The “still” epochs chosen for the analysis are behaviorally similar, facilitating the interpretation of pharmacological effects on qEEG features. Without overt behavioral effects, the unavoidable variability in drug exposures transforms from being a source of error in dosing regimes into an informative quantitative variable when bioanalytical determination is deployed. We have found that differences in exposure partially explain the variability in EEG measures. Establishing a correlation within the limited range of exposures obtained in single-dose experiments highlights the value of qEEG as a highly sensitive pharmacological biomarker.

Diazepam EIB disruption is concentration-dependent, and it can be measured in the most prominent EEG features: β, θ, and γ oscillations, and aperiodic 1/f activity. β Band amplitude and AUC correlate to diazepam exposure in the parietal cortex. Although not significant in the auditory and frontal cortices, the β band also follows a positive tendency in these regions. The θ band modal frequency positively correlates to diazepam exposure in both the auditory and parietal cortices. Our data points to a faster θ band up until ∼25 ng/ml, at which point there is no θ peak detected. We speculate that with increasingly higher concentrations of diazepam, the θ peak disappears in favor of the larger β oscillatory power. Similarly, the low-γ AUC, which negatively correlates to diazepam exposure in the auditory cortex, may progressively decrease toward vehicle levels as the concentration of diazepam increases because low-γ is being overridden by increasingly larger β band activity. Interestingly, the aperiodic slope does not correlate to diazepam exposure. However, modal peak frequencies do positively correlate to exposure in some oscillatory bands, which may be enabled by the drop in broadband power driven by the steepening of the slope. Although both the slope and offset of the aperiodic component are changed under diazepam, the only parameter that tends to correlate to exposure is the aperiodic knee. Although the overall tendency for the knee is to increase under diazepam, in the specific case of the parietal cortex, the knee negatively correlates to exposure. The aperiodic knee frequency informs on neuronal timescales, which are region-dependent following a rostrocaudal gradient in humans (Gao et al., 2020). Our results further support such regional specificity, while shedding light on the temporal dynamics induced by diazepam.

Similarly, MK-801 disruption of EIB is also concentration-dependent, as evidenced by the correlation between exposure and the most salient EEG features: aperiodic slope, θ band activity, γ band activity, and HFO. Regarding the aperiodic component, the slope positively correlates to exposure. It is worth noting that for lower doses, the aperiodic slope is smaller than in the vehicle. But as the concentration increases, the slope becomes increasingly larger, surpassing vehicle levels at ∼4 ng/ml. A similar pattern is observed in the amplitude and AUC of low-γ in the auditory cortex; although at low exposures low-γ under MK-801 is larger than under vehicle conditions, increasingly higher exposures decrease the difference, even reverting it at ∼5 ng/ml. These concentration-dependent shifts in the PS features may be a reflection of MK-801 dual mechanism. At low concentrations, MK-801 causes psychotomimetic responses, while at higher doses it has an anesthetic effect.

Mid-γ AUC and amplitude positively correlate to exposure in the auditory cortex. This was expected as mid-γ increases are the most replicated findings of NMDAR antagonist and in our data the auditory cortex is the region in which the mid-γ is significantly larger than in the vehicle condition. Surprisingly, we found that some parameters correlate with MK-801 exposures but do not reach statistically significant differences compared with vehicle, such as θ AUC or the low-γ amplitude and AUC. In this case, the correlation with exposures suggests that higher doses could modulate these parameters significantly, although behavioral confounds at higher doses would come into play.

Lastly, MK-801 exposure correlates to changes in γ and HFO modal frequencies. Overall, increasingly higher exposure levels of MK-801 lead to faster modal frequencies, except for low-γ. Low-γ modal frequency in the frontal cortex negatively correlates to exposure, indicating a slowing down of frequencies <40 Hz. Conversely, in the parietal and frontal cortex, the high-γ and HFO positively correlate to exposure, indicating that MK-801 leads to a speeding up of higher frequencies. These seemingly contradictory tendencies only emphasize the need to understand the EEG spectrum as a whole.

In conclusion, we have demonstrated and quantified the opposing effects on EIB of the NMDAR antagonist MK-801 and the GABAAR modulator diazepam through the decomposition of the qEEG PS into the oscillatory periodic and the 1/f aperiodic components. Furthermore, we have established a correlation between drug exposure and several parameters of the periodic and aperiodic components of the qEEG signal. As other neurotransmitter systems modulate the EIB, it remains to be explored what the decomposition of the qEEG PS signal will reveal about their mechanism. This is important because of the highly complex interplay between the glutamatergic and GABAergic systems that maintain EIB.

Because of the translatability of the techniques (EEG and plasma sampling), it would be interesting to compare the results of this rodent work to human EEG. Ultimately, the quantification of translational features linked to EIB would constitute a valuable biomarker for the preclinical and clinical development of therapeutic treatments aimed at correcting excitatory-inhibitory imbalances.

## Synthesis

Reviewing Editor: William Stacey, University of Michigan Decisions are customarily a result of the Reviewing Editor and the peer reviewers coming together and discussing their recommendations until a consensus is reached. When revisions are invited, a fact-based synthesis statement explaining their decision and outlining what is needed to prepare a revision will be listed below. The following reviewer(s) agreed to reveal their identity: Richard Gao, Poortata Lalwani.

The reviewers and I have discussed the article and find it to be an interesting and useful evaluation. There are a couple of important questions, and several other suggestions for improvement:

1. Statistics: There were some questions regarding the choice of statistical methods. First, it appears you aggregate across cohorts and different equipment but do not control for that or measure whether that affected the results. Seconds, please ensure that appropriate multiple comparisons corrections have been done and refrain from (or justify) using one-tailed tests. If the authors intend to still collapse across cohorts especially in the exposure analysis (as they have) - please include appropriate regressors, or show lack of cohort effect, or that the results are unaffected by restricting it to cohort 2 and including cohort 1 simply helps with the strength.

2. Additional analysis: There are age and task related differences in 1/f slope as pointed by the authors in the discussion. Since the authors have a still vs. moving and old vs. young data it would be powerful to investigate and include a replication of these age and task effects. It will be particularly impactful to investigate how EIB manipulations are affecting various EEG measures across age-cohorts and task-conditions. If you do not do this (we do not explicitly require additional experiments), you should perform at least a correction for the age- and cohort-effect and show that the results remain similar (even if significance drops with sample size). These results should also be reported.

3. page 13: regarding the statement “this suggests that NMDAR modulation may have different effects on the EIB depending on brain region” - since the mechanistic manipulation is known here, i.e. NMDAR antagonist decreases excitation, which is actually the gold standard experiment, shouldn’t the interpretation be instead that shifting EIB via NMDA modulation may have different effects on the consequent circuit dynamics and neurophysiological readout (i.e., LFP) depending on the brain region?

4. The observation regarding knee changes under MK0801 (and the investigation of this feature in general) is very interesting. Gao et al 2020 eLife interpreted the knee parameter, which could potentially link these results to another growing body of literature on neural timescales. These data, interpreted under that light, would be of high interest to that community, especially given NMDA’s special role in maintaining longer timescale computations involved in working memory, decision making, etc. On that note, the knee is typically very non-normally distributed. It looks like all the statistical tests are performed on Gaussianized features already, but it may be more informative to plot the log-transformed knee, or converted to units of frequency as in Donoghue et al 2020 or time constant as in Gao et al 2020, for Fig 3C.

5. Fig 7 & 8: for the plasma concentration vs. spectral feature analysis, it would make more sense to correlate change in spectral feature (e.g., delta slope) vs. plasma concentration. Perhaps this was already done and we missed it, but the figures imply that correlation was computed just on the post-exposure measures. Legend within the figure for the two types of markers would also be informative.

6. regarding the discussion on why there may be region specific variations (page 23-24), it’s worth briefly discussing that there are meaningful differences in circuitry between the regions in question, in terms of cell type (and their associated ion channels), density of various synaptic receptors, as well as connectivity, all of which would contribute to a differential response towards a blanket pharmacological intervention. For us, this is a much more likely (and interesting) explanation than the simple one provided (on page 24).

7. Presentation : As there are several measures of interest (slope, intercept, bands, regions, drugs etc.) it is difficult to keep track of all the questions and their respective answers. We recommend including a summary results table. The results section is relatively hard to follow and dense - please try restructuring and rephrasing it. Consider separating some results into supplementary and main text.

8. There are several research articles investigating role of EIB in various EEG measures that have (and some have not) been included in the discussion. Claims of novelty may be difficult, but also are not really necessary. However, it is critical to embed the findings more thoroughly in past work (what is supporting or contrasting) rather than simply summarizing what is known and what was found again. A recent review provides a summary of these: https://www.nature.com/articles/s41398-022-02218-z

Minor comments:

- page 4: oscillatory & periodic are typically used synonymously?

- page 4: typo: NMDAR antagonist?

- page 10: typo in Lorentzian function formula, missing a bracket in the denominator

- page 13: typo, first sentence, analysis of the periodic component

- page 14 middle: typo, to conclude the analysis of the aperiodic component

- Fig 4A: it may be informative to label the unit for Aperiodic Slope, which here I guess is decibels/Hz? This is just to clarify the unexpected scale (10 to 30), as the exponent typically varies in the range of 1 to 4.

- Fig 6: while the color scheme is clear, the inconsistency in x-axis grouping (A & D vs the rest) introduces some confusion, especially when the 2 columns of the same row don’t match (like D)
