# Supplementary material for: Glutamatergic and GABAergic Receptor Modulation Present Unique Electrophysiological Fingerprints in a Concentration-Dependent and Region-Specific Manner
Source: eNeuro. 2023 Apr 13;10(4):ENEURO.0406-22.2023. doi: 10.1523/ENEURO.0406-22.2023 (PMC10124153; doi:10.1523/ENEURO.0406-22.2023)
Supplement: Extended Data Table 2-1 — Statistical table. All tests were performed on normally distributed data. Shown are (a–l) Tukey’s multiple comparisons test for significant ANOVAs and (m, n) unpaired t test with Welch’s correction. Download Table 2-1, DOCX file. [file enu-eN-NWR-0406-22-s09.docx]

| **Ref** | **p-values** | **95% CI of diff** |
| --- | --- | --- |
| a)  Fig. 4A | Vehicle vs. MK-801: 0.004  Vehicle vs. Diazepam: <0.001  MK-801 vs. Diazepam: <0.001 | -1.226 to -0.215  -2.226 to 1.215  -1.505 to -0.495 |
| b) Fig. 4B | A1 - Vehicle vs. MK-801: 1.000  A1 - Vehicle vs. Diazepam: 0.001  A1 - MK-801 vs. Diazepam: <0.001  PAR - Vehicle vs. MK-801: 0.082  PAR - Vehicle vs. Diazepam: <0.001  PAR - MK-801 vs. Diazepam: 0.103  FRC - Vehicle vs. MK-801: 0.131  FRC - Vehicle vs. Diazepam: <0.001  FRC - MK-801 vs. Diazepam: 0.240 | -0.853 to 1.063  -2.368 to -0.452  -2.473 to -0.557  -1.855 to 0.061  -2.721 to -0.805  -1.824 to 0.092  -1.791 to 0.125  -2.534 to -0.618  -1.701 to 0.215 |
| c) Fig. 4C | Vehicle vs. MK-801: <0.001  Vehicle vs. Diazepam: <0.001  MK-801 vs. Diazepam: <0.001 | -1.368 to -0.373  -2.368 to -1.373  -1.497 to -0.503 |
| d) Fig. 6A | A1 - Vehicle vs. MK-801: 0.998  A1 - Vehicle vs. Diazepam: 0.014  A1 - MK-801 vs. Diazepam: 0.002  PAR - Vehicle vs. MK-801: 1.000  PAR - Vehicle vs. Diazepam: 0.001  PAR - MK-801 vs. Diazepam: 0.002  FRC - Vehicle vs. MK-801: 0.556  FRC - Vehicle vs. Diazepam: 0.148  FRC - MK-801 vs. Diazepam: 0.996 | -1.429 to 0.911  0.182 to 2.522  0.441 to 2.780  -1.055 to 1.284  0.546 to 2.885  0.432 to 2.771    -0.462 to 1.878  -0.173 to 2.166  -0.881 to 1.458 |
| e) Fig. 6A | A1 - Vehicle vs. MK-801: 0.995  A1 - Vehicle vs. Diazepam: 0.044  A1 - MK-801 vs. Diazepam: 0.257  PAR - Vehicle vs. MK-801: 0.543  PAR - Vehicle vs. Diazepam: 0.006  PAR - MK-801 vs. Diazepam: 0.500  FRC - Vehicle vs. MK-801: 0.605  FRC - Vehicle vs. Diazepam: 0.729  FRC - MK-801 vs. Diazepam: 1.000 | -1.675 to 2.803  0.035 to 4.514  -0.529 to 3.950    -0.870 to 3.608  0.544 to 5.023  -0.825 to 3.654  -1.315 to 3.164  -1.069 to 3.409  -1.994 to 2.485 |
| f) Fig. 6B | Vehicle vs. MK-801: 0.012  Vehicle vs. Diazepam: <0.001  MK-801 vs. Diazepam: 0.001 | -1.309 to -0.138  -2.309 to -1.138  -1.585 to -0.415 |
| g) Fig. 6B | Vehicle vs. MK-801: 0.055  Vehicle vs. Diazepam: <0.001  MK-801 vs. Diazepam: 0.001 | -1.243 to 0.011  -2.243 to -0.989  -1.627 to -0.373 |
| h) Fig. 6D | A1 - Vehicle vs. MK-801: 0.001  A1 - Vehicle vs. Diazepam: 0.998  A1 - MK-801 vs. Diazepam: 0.011  PAR - Vehicle vs. MK-801: 0.220  PAR - Vehicle vs. Diazepam: 0.979  PAR - MK-801 vs. Diazepam: 0.819  FRC - Vehicle vs. MK-801: 0.163  FRC - Vehicle vs. Diazepam: 0.484  FRC - MK-801 vs. Diazepam: 0.999 | -3.389 to -0.566  -1.725 to 1.097  0.253 to 3.075    -2.526 to 0.296  -1.858 to 0.964  -0.743 to 2.079    -2.593 to 0.230  -2.313 to 0.509  -1.132 to 1.691 |
| i) Fig. 6E | A1 – PAR: < 0.001  A1 – FRC: 0.001  PAR – FRC: 0.001 | -2.627 to -1.422  -1.627 to -0.422  0.398 to 1.603 |
| j) Fig. 6E | A1 – PAR: < 0.001  A1 – FRC: 0.007  PAR – FRC: 0.001 | -2.440 to -1.195  -1.440 to -0.195  0.378 to 1.623 |
| k) Fig. 6F | A1 - Vehicle vs. MK-801: 0.147  A1 - Vehicle vs. Diazepam: 0.988  A1 - MK-801 vs. Diazepam: 0.016  PAR - Vehicle vs. MK-801: <0.001  PAR - Vehicle vs. Diazepam: <0.001  PAR - MK-801 vs. Diazepam: 0.988  FRC - Vehicle vs. MK-801: <0.001  FRC - Vehicle vs. Diazepam: <0.001  FRC - MK-801 vs. Diazepam: 0.021 | -2.145 to 0.170  -0.825 to 1.490  0.162 to 2.477  -3.009 to -0.694  -0.688 to 1.627  1.164 to 3.478  -3.710 to -1.396  -0.825 to 1.490  1.728 to 4.043 |
| l) Fig. 6F | A1 - Vehicle vs. MK-801: 0.415  A1 - Vehicle vs. Diazepam: 0.986  A1 - MK-801 vs. Diazepam: 0.064  PAR - Vehicle vs. MK-801: 0.002  PAR - Vehicle vs. Diazepam: 0.900  PAR - MK-801 vs. Diazepam: <0.001  FRC - Vehicle vs. MK-801: <0.001  FRC - Vehicle vs. Diazepam: 0.968  FRC - MK-801 vs. Diazepam: <0.001 | -2.086 to 0.407  -0.878 to 1.615  -0.039 to 2.455    -2.981 to -0.488  -0.727 to 1.767  1.008 to 3.501    -3.558 to -1.065  -0.878 to 1.615  1.433 to 3.927 |
| m) text | Unpaired t-test with Welch’s correction 0.0430 | -3.915 to -0.07968 |
| n) text | Unpaired t-test with Welch’s correction  0.0015 | -32.13 to -10.99 |
